# Sensor-Based Real-Time Condition Monitoring and Three-Level Fault Pre-Warning for Cryogenic Dewar Systems

**DOI:** 10.3390/s26216799

**Published:** 2026-10-27

**Authors:** Hao Yan, Maozheng Chen, Jun Ma, Yanling Liu, Kai Wang, Liang Cao, Xiaofei Li

**Affiliations:** 1 Xinjiang Astronomical Observatory, Chinese Academy of Sciences, Urumqi 830011, China; yanhao@xao.ac.cn (H.Y.); majun@xao.ac.cn (J.M.); liuyanling@xao.ac.cn (Y.L.); wangkai@xao.ac.cn (K.W.); caoliang@xao.ac.cn (L.C.); lixf@xao.ac.cn (X.L.); 2 University of Chinese Academy of Sciences, Beijing 100049, China; 3 Xinjiang Key Laboratory of Microwave Technology, Urumqi 830011, China

**Keywords:** condition monitoring, fault diagnosis, predictive maintenance, anomaly detection, online clustering, AR(2) extrapolation, sensor data processing, cryogenic systems

## Abstract

Cryogenic dewars housing low-noise amplifiers are safety-critical: a slow temperature rise can indicate loss of vacuum or insufficient cooling and may escalate into hard failure within hours to days, yet fixed-threshold monitoring gives too little lead time and no explanation of the alarm cause. This paper proposes a sensor-based real-time condition monitoring and three-level fault pre-warning method, demonstrated on real two-stage temperature and vacuum records from the L, C and Q band dewars of a 26 m radio telescope. Sliding-window smoothing precedes online k-means, which assigns each frame to one of four states (steady, rising, dropping, ambient). In a steady state, an AR(2) model extrapolates the trend, and a finite state machine issues yellow, orange, and red warnings according to the rate of change.The vacuum reading serves as an auxiliary criterion for joint warning. On over 20 million real frames (bad-point rate up to 37.5%), it raises precision—measured as the agreement of the warning frames with the method’s own event list rather than as an independent fault detection accuracy—from 0.0486 to 0.2051 on C and from 0.0009 to 0.2484 on Q in a same-protocol comparison on the calibration (early) segment, not a held-out test. It also lowers the out-of-zone warning-frame fraction, attains a pre-warning window recall of 0.93–1.00, and runs at about 1.8 μs per frame ($5.6\times 10^{5}$ frames/s), fast enough for real-time embedded use. Continuous monitoring captured only steady or mildly trending segments, with no hard fault triggered.

## 1. Introduction

The core devices of a cryogenic system, such as the cryogenic low-noise amplifier, are encapsulated in a vacuum dewar [1,2,3] and maintained at a cryogenic operating point of about 12–20 K by a refrigerator [4]. Cryogenic dewar systems of this kind are deployed not only in radio astronomy but also in quantum computing, medical imaging and superconducting facilities, and they share a common trait: they are safety-critical, expensive to repair, and their performance degrades long before a visible breakdown. Once the internal vacuum of the dewar drops or the cooling power becomes insufficient, the dewar temperature will slowly rise and evolve into a hard failure at ambient-temperature scale within hours to days [5]. This directly causes the loss of the cryogenic stage and thereby degrades the sensitivity of the hosted instrument [6,7] and the long-term availability of the facility [6]. Therefore, issuing a credible pre-warning before the temperature enters the irreversible range has direct engineering value for equipment safety and operational continuity, which is exactly the objective of sensor-based condition monitoring and predictive maintenance.

In the field of equipment condition monitoring, statistical process control methods (such as EWMA [8,9] and Shewhart control charts [10]) can analytically control the false alarm rate, but they assume a stationary process and have limited adaptability to trend-like slow changes [11,12] and multimodal distributions [13,14,15]. Autoregressive models (ARIMA and its low-order variants) can characterize the inertial properties of temperature with very few parameters [16,17,18] and are suitable for real-time embedded deployment [19,20], yet they usually only do point prediction [21,22] and do not provide interpretable fault-pre-warning semantics [21,23]. K-means++ [24] and its online incremental variant [25] can perform unsupervised partitioning of equipment operating states [26,27], but generic cluster centers lack physical interpretability specific to dewar thermal states [28,29]. Among them, the four-state K-means++ partitioning for dewar temperature was proposed by the authors’ prior work [30], and this paper extends it into a real-time pre-warning framework. Isolation forest [31] is an unsupervised batch method and therefore needs no labels, but it presupposes that the training set covers representative samples of the normal operating regime, and its anomaly score is a relative measure that carries no explicit physical or state semantics. A recent variant of it builds the isolation trees by merging nodes under a minimum spanning tree and scores an object by tree height together with the distance to its nearest point, which improves classification accuracy over the base method [32]. It inherits exactly the two properties that exclude it from the present setting. The forest, and in particular the spanning tree, has to be built from the complete observation history and rebuilt as new data arrive. The target here, by contrast, is a per-frame O(H) online loop on an embedded front end with no training phase and no full-history rescan. Its output is still an anomaly score that carries neither the state semantics nor the forecast horizon from which a graded yellow–orange–red warning with an explicit reason can be derived. Autoencoders [33] likewise need no labels, but they depend on a substantial training set, an offline training phase and periodic retraining, and their reconstruction error admits no direct physical interpretation. LSTM-based detectors [34] require labeled anomaly sequences in their supervised formulation, whereas in their unsupervised formulation, such as forecasting-error approaches, they require at least a sufficiently long clean history from which a reliable baseline can be established [35,36]. Recent comprehensive evaluations further indicate that the practical applicability of such detectors is governed by method-specific prerequisites—representativeness of the training set, offline training budget and retraining cadence—rather than by detection accuracy alone [37]. These methods perform excellently on general time-series anomaly detection [38,39]. Yet in the present setting the above prerequisites cannot all be satisfied simultaneously during routine operation, and their black-box nature is unfavorable for interpretable auditing in high-reliability scenarios [40]. However, none of the above methods offers an end-to-end real-time pre-warning solution tailored to the characteristics of dewar multi-band monitoring data—“long steady state, sparse faults, frequent bad points”. Meanwhile, existing engineering monitoring mostly relies on fixed thresholds [41,42], which often alarm only after a fault occurs with insufficient lead time [41,42], and it is difficult to explain the alarm cause in real time [43].

To fill this gap, this paper takes the real multi-sensor monitoring data of the L, C, and Q band cryogenic dewar systems of a 26 m radio telescope as a representative case study and proposes a sensor-based real-time condition monitoring and three-level fault pre-warning method. First, online k-means clusters the temperature into four categories—steady-state, rising, dropping, ambient (the four-state partitioning follows the authors’ prior work [30]). Then, an AR(2) autoregressive model extrapolates the steady-state trend; then, combined with the temperature change rate and a finite state machine (FSM), it gives three levels of warnings: yellow, orange, and red. The vacuum degree attached to the L band is introduced as an auxiliary criterion to realize multi-parameter joint warning.

The main contributions of this paper are as follows. (1) At the method level, a lightweight framework combining online k-means four-state classification with AR(2) extrapolation is proposed, which balances steady-state baseline maintenance with online adaptation to event states. A three-level warning mechanism centered on the identification factor, extrapolation breakthrough, and FSM is designed, with traceable warning reasons. (2) At the data level, all experiments are end-to-end rerun on real dewar monitoring data, with no simulation on the evaluation side. The influence of the same-source nature of fault labels and warnings is candidly disclosed in both directions: it biases recall-type metrics upward, and it also fixes the meaning of the reported precision and F1, which measure agreement with this method’s own event list rather than independent fault detection accuracy. (3) At the performance level, on the three-band data, the precision is improved by 1–2 orders of magnitude over the fixed-threshold and EWMA baselines, the recall within the pre-warning window reaches 0.93–1.00, and the per-frame processing takes about 1.8 μs (about $5.6\times 10^{5}$ frames per second).

The rest of this paper is organized as follows. Section 2 gives the method details (including the system framework, symbol definition, parameter configuration, and the online main loop). Section 3 describes data acquisition and preprocessing. Section 4 reports the experimental results. Section 5 discusses the validity boundary imposed on the evaluation metrics by the same-source alarm-log labels, the reason why the L band produced no orange or red warnings, the advantages and limitations of the proposed method, and how the method compares with SPC control charts and with black-box machine learning detectors. Section 6 concludes the paper and outlines directions for future research.

## 2. Method

### 2.1. System Framework

**Figure 1:**
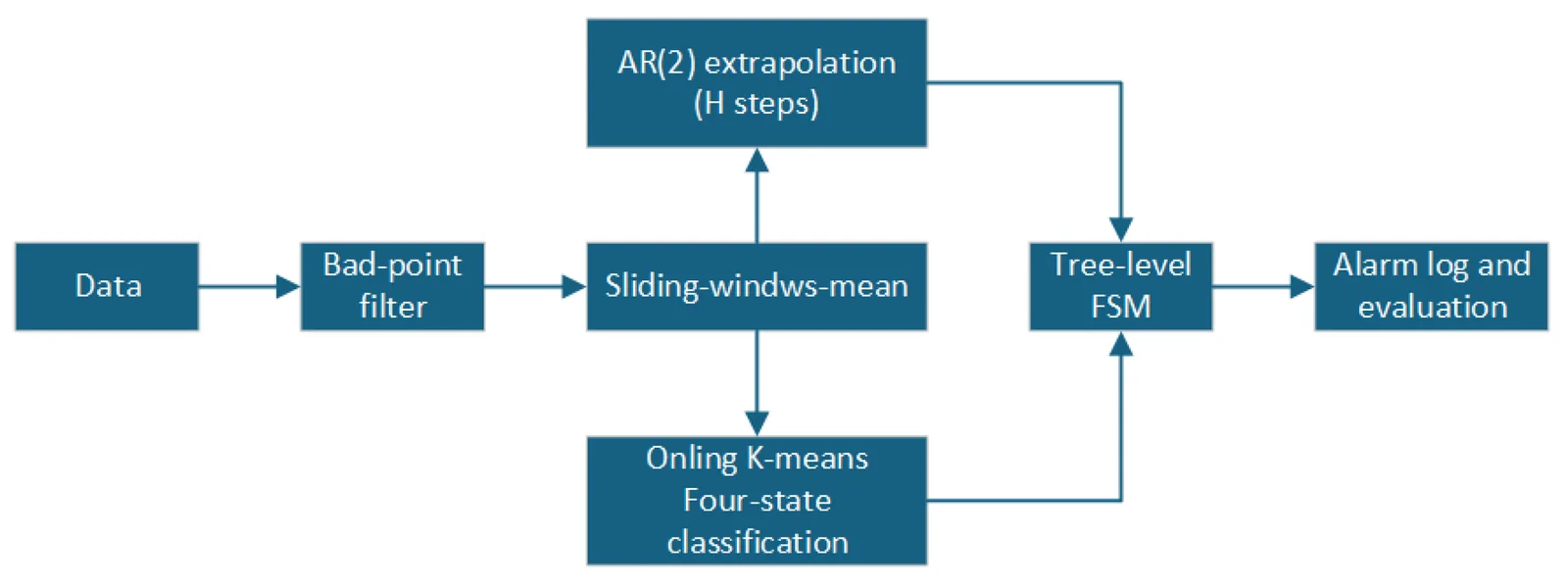
System framework: after filtering and smoothing, the dewar monitoring data are classified and extrapolated in parallel, driving the three-level warning FSM.

This method is designed with the goals of being “lightweight, white-box, real-time”, and is an overall single data flow serial structure, as shown in Figure 1. The dewar monitoring CSV first goes through the bad-point filtering of Section 3.2 and the sliding-window smoothing of Section 3.3 to obtain the smoothed observation $\left({\bar{T}}_{1},{\bar{T}}_{2}\right)$ of each frame. This observation is then fed in parallel into two independent computation branches—one is online k-means four-state classification (giving the current thermal-state semantics), the other is AR(2) temperature extrapolation (giving the predicted value of the future *H* steps). The outputs of the two branches, together with the identification factor (window mean slope winSlope) computed from the same window, jointly drive the three-level warning finite state machine (FSM), and finally result in yellow/orange/red three-level alarms written to the log for offline evaluation.

This structure has two points worth noting. First, classification and extrapolation are “parallel but decoupled”: classification cares about “what state are we in at this moment”, while extrapolation cares about “where will it go following this inertia”. The two are complementary rather than redundant so that the warning knows both the status and the trend. Second, all operations are frame-by-frame, fixed-complexity lightweight operations (nearest neighbor, second-order recursion, mean), with no dependence on a training phase. It can therefore run continuously on the embedded front end; the per-frame overhead is in Section 4.5. Section 2.3, Section 2.4 and Section 2.5 expand each module in turn, and Section 2.6 summarizes the main loop with pseudocode.

### 2.2. Symbol Definition

For ease of subsequent presentation, Table 1 summarizes the main symbols used throughout the paper and their values (only the report gate *N* is selected by the ablation in Section 4.2; the other values are implementation defaults). Temperatures are uniformly in K. Because the sampling intervals differ across bands, all quantities involving “time length” (window *W*, extrapolation horizon *H*, pre-warning window relWin, consecutive-frame gate *N*) use “frame” as the unified unit, avoiding wrongly generalizing one band’s second-level conversion to all bands (see Section 3.1). Symbols are not redefined when referenced below.

### 2.3. Online k-Means Four-State Classification

The goal of this module is to assign each frame’s smoothed observation $\left({\bar{T}}_{1},{\bar{T}}_{2}\right)$ interpretable “thermal-state semantics”, providing context for the subsequent warning. The four-state partitioning and the offline cluster-center initialization follow the authors’ prior work [30] (Research on Receiver Dewar Temperature Monitoring and Fault Prediction Based on K-means++): the dewar temperature is induced by physical evolution into four states—steady state under normal refrigeration (NORMAL), slow warming (RISE), slow cooling (DROP), and ambient after shutdown/vacuum loss (AMBIENT). NORMAL is a steady state and not a cooling transient: it is the low-temperature equilibrium held by the refrigeration system, whereas DROP is the slow cooling transient leading away from it. On the basis of the prior four-state K-means++ partitioning, this paper further introduces online incremental update and a three-level warning mechanism (Section 2.5), forming a complete real-time precursor-warning framework. To respect the method origin, this section explicitly marks its inheritance from [30] to avoid confusion with the original method. Four states are chosen instead of two to distinguish “still healthy fluctuation” (NORMAL) from “already showing a warming trend” (RISE)—the latter is precisely the fault precursor that requires a higher level of attention; DROP and AMBIENT are used to characterize outliers such as maintenance or vacuum loss.

Offline center initialization. Because the steady-state segment has a known and clearly bounded temperature distribution, this paper follows [30] and does not rely on randomly initialized K-means++, but directly cuts the temperature into four states in the steady-state segment using physical thresholds, taking the within-state sample mean as the cluster center $c_{s}=\left(T_{1},T_{2}\right)$. This is a physically initialized center: the state of each training frame is determined by a physical threshold rather than by iterative clustering, and the center is the mean of the frames assigned to that state. It is therefore not a K-means++ seeding in the sense of [24]—no D^2^-weighted probabilistic seeding is performed—and we do not use that name for it. The term “online k-means” is used throughout for the resulting procedure because the nearest-neighbor assignment of Equation (1) and the incremental center update of Equation (2) run online; the initialization is a supervised, physically defined step that precedes them. These offline-calibrated four-state centers, together with the AR(2) coefficients of Section 2.4 and the physical thresholds and residual standard deviation σ of Section 2.5, are listed as numerical parameters band by band (see the per-band coefficient table). For the L band, NORMAL center $c_{0}\approx \left(53.94,\;12.69\right)$ K, RISE center $c_{1}\approx \left(59.66,\;13.62\right)$ K, DROP center $c_{2}\approx \left(60.42,\;13.67\right)$ K, AMBIENT center $c_{3}\approx \left(319.81,\;12.49\right)$ K, and AR(2) coefficients $a_{1}=0.3881,\;a_{2}=0.4602,\;c=1.8448,\;\sigma =0.1193$. Each center has a clear physical meaning: for example, the NORMAL center is the steady-state operating point of each band for each band (L about 12.69 K, C about 16.32 K, Q about 17.82 K), rather than a non-interpretable geometric centroid.

**Figure 2:**
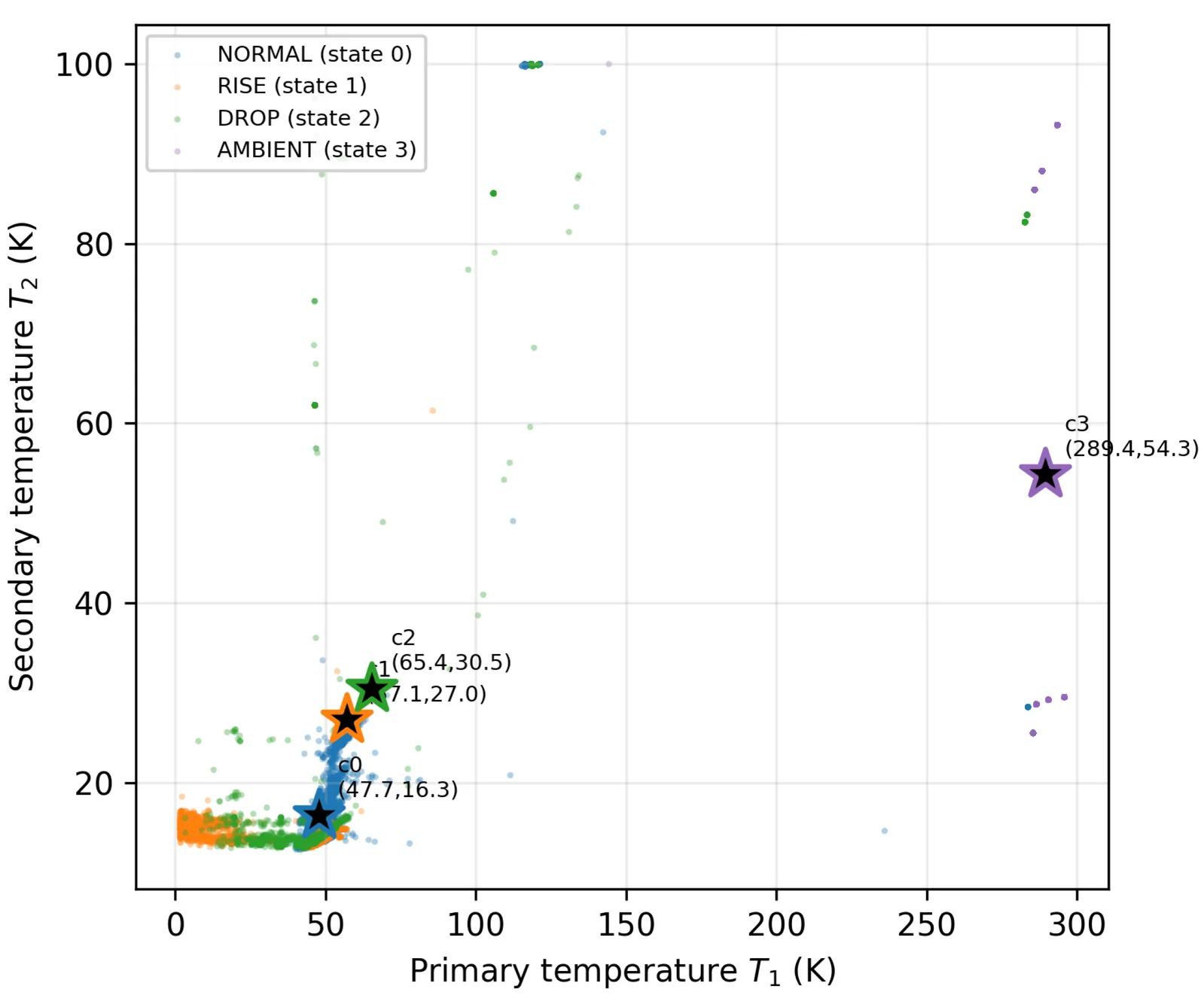
Online k-means four-state classification on real C-band data in the $T_{1}$–$T_{2}$ feature plane. Points are colored by assigned state (NORMAL/RISE/DROP/AMBIENT); starred markers are the four calibrated cluster centers $c_{s}$.

Note on the L-band AMBIENT center. The L-band AMBIENT center calls for a comment since its two coordinates look mutually inconsistent: ${\bar{T}}_{1}\approx 319.8$ K is far above the ambient threshold while ${\bar{T}}_{2}\approx 12.49$ K is essentially the NORMAL level. This center is the mean of only 21 frames, and those frames are not a warm-up episode. They are isolated single-frame spikes of the first-stage sensor; the frames 32 s before and after show a normal ${\bar{T}}_{1}$ of about 48–52 K with ${\bar{T}}_{2}$ near 12 K and the vacuum reading at a healthy value of 0.005 throughout. A genuine warm-up looks entirely different—one such episode in the same record has ${\bar{T}}_{1}=293.1$ K, ${\bar{T}}_{2}=290.3$ K and a vacuum reading of 2490. The spikes are thus a sensor artifact, not a thermal state, and their ${\bar{T}}_{2}$ stays at the NORMAL level because the second stage never warmed. Genuine warm-up frames do occur, but the bad-point rule of Section 3.2 substitutes them in place and keeps them out of the fitting windows because that rule accepts $T_{2}$ values of only up to 100 K whereas the 319.8 K spikes fall inside the accepted $T_{1}$ range and survive. Excluding such frames from the offline fitting does not remove them from the online record. A genuine warm-up is declared on the first-stage channel while both channels are still inside their accepted ranges, and how far the 100 K bound can influence the reported warning level is quantified in Section 3.2. For contrast, the C-band AMBIENT center shown in Figure 2 does lie in the high-temperature direction (${\bar{T}}_{2}\approx 54.3$ K), and the Q band has no AMBIENT frames at all. This does not affect classification since AMBIENT is excluded from nearest-neighbor assignment and is triggered only by the physical hard decision given below.

Online nearest-neighbor classification. In the online stage, each frame assigns $\left({\bar{T}}_{1},{\bar{T}}_{2}\right)$ to the cluster with the smallest Euclidean distance:

(1) $$s^{*}=\arg\min_{s}{∥\left({\bar{T}}_{1},{\bar{T}}_{2}\right)-c_{s}∥}^{2}.$$

Online incremental update (extension of this paper). The prior work [30] obtains fixed four-state centers by offline K-means++. On this basis, this paper adds an online incremental update, enabling the centers to adapt to slow environmental drift (such as seasonal temperature drift and refrigerator aging). This is a real-time extension of the prior work (the core of online k-means):

(2) $$c_{s^{*}}\leftarrow c_{s^{*}}+\rho \;\left(\left({\bar{T}}_{1},{\bar{T}}_{2}\right)-c_{s^{*}}\right),\;\rho =0.01.$$

The learning rate ρ=0.01 is taken small, so that the center only changes slowly with long-term drift and is not violently dragged by a single-frame event.

Two key constraints. First, the NORMAL center is frozen and not updated, to preserve the “healthy baseline” from being contaminated by warming events— otherwise, once a fault-warming sample enters the window, the steady-state baseline would be raised, causing distortion of the subsequent extrapolation threshold. Second, the AMBIENT state does not participate in nearest-neighbor classification but is triggered only by a physical hard decision (${\bar{T}}_{2}>T_{2}^{\mathrm{amb}}$, or ${\bar{T}}_{1}>T_{1}^{\mathrm{amb}}$ when vacuum is enabled, where $T_{2}^{\mathrm{amb}}$ is the ambient threshold). This is because AMBIENT and NORMAL may be adjacent in the feature space. If nearest-neighbor merging were allowed, high-temperature frames could be misclassified as ambient, masking a real hard jump. Changing it to a hard decision prevents such “illegal-jump false red” and leaves the capture of the NORMAL↔AMBIENT abrupt change to the FSM legality check in Section 2.5.

Figure 2 visualizes the above four-state partitioning on real C-band data: each point is a frame’s smoothed observation $\left({\bar{T}}_{1},{\bar{T}}_{2}\right)$ colored by its assigned state, and the black-bordered stars mark the four offline-calibrated cluster centers $c_{s}$ from Equation (1). The four states occupy clearly separated regions in the $T_{1}$–$T_{2}$ plane—NORMAL sits at the cool steady-state operating point, RISE and DROP spread along the warming/cooling directions, and AMBIENT is isolated at the far high-temperature corner—confirming that the physical-threshold initialization yields geometrically and physically meaningful clusters rather than arbitrary partitions. This separation is precisely why the nearest-neighbor assignment can supply reliable thermal-state context to the downstream warning.

### 2.4. AR(2) Temperature Extrapolation

The goal of this module is to “look ahead”: based on the observed steady-state temperature, predict whether the healthy upper bound will be breached in the future, thus giving a fault precursor. The dewar secondary temperature has obvious inertia (damping caused by heat capacity)—the warming is a gradual change rather than an instantaneous jump. A linear extrapolation using only “the difference of the last two frames” therefore over- or under-estimates the trend, while an autoregressive model can use a longer history to characterize this inertia.

Model fitting. Only the NORMAL steady-state segment $T_{2}$ series is used to fit the second-order autoregressive model offline:

(3) $$x_{t}=a_{1}x_{t-1}+a_{2}x_{t-2}+c.$$

The coefficients $a_{1},a_{2},c$ are obtained by least squares, and the residual standard deviation σ is given synchronously (used as the confidence-band width of the subsequent orange threshold). Fitting is restricted to the NORMAL segment, ensuring that the model expresses the “inherent inertia under healthy operation” rather than mixing in anomalous trajectories after faults. Taking the L band as an example, the fit gives $a_{1}+a_{2}=0.8483<1$, i.e., the process is stationarily convergent, with a steady-state value of about 12.16 K (see Section 5), of the same order as the extrapolation and the measured center.

Multi-step extrapolation. AR(2) is a second-order recursive model; multi-step extrapolation needs only the most recent two frames as initial values. This paper uses the current frame $x_{n}$ smoothed by the sliding window of Section 3.3 (length W=50 frames) and its previous frame $x_{n-1}$ as the recursion initial values (*n* is the current frame index). It then recursively obtains the prediction of the *h*-th future step by Equation (4):

(4) $${\hat{x}}_{n+h}=a_{1}{\hat{x}}_{n+h-1}+a_{2}{\hat{x}}_{n+h-2}+c,\;h=1,\ldots ,H.$$

This paper takes the extrapolation horizon H=300 frames. Its physical meaning is “how far to look ahead”. Because the sampling interval differs per band, the same 300 frames denote 5.17 h on L, 50 min on C and 2.83 h on Q in the later segment and 2.67–5.17 h on L in the early segment where that band is bimodal. The larger *H*, the wider the pre-warning window and the more lead time, but the more sensitive to extrapolation error. This paper takes 300 frames aligned with the pre-warning window relWin, set by the sensitivity study of Section 4.3, so that “extrapolation breakthrough” corresponds to “5.17 h (L), 50 min (C) or 2.83 h (Q) before the fault start”, consistent with the pre-warning window recall protocol used in the experiments.

Why AR(2) rather than linear extrapolation at a long horizon. Linear extrapolation assumes a constant slope and carries no bound, so its error grows without limit with the horizon. The second-order memory of AR(2) can express the inflection behavior of “warming fast then slow” or “approaching steady”, which better fits dewar thermodynamics, and its stationary root ($a_{1}+a_{2}<1$) damps the forecast. To verify this at the horizon that was actually used, the baseline comparison in Section 4.4 adds a linear extrapolation that forecasts the same horizon H=300, and the multi-step errors of both rules are measured at h=1,…,300 in Table 2. The advantage of AR(2) is real but is confined to long horizons—at h≤10, the linear rule is in fact the more accurate of the two. The claim made here is therefore deliberately narrower than “AR(2) is uniformly superior”: it is at H=300, the horizon used for warning, that the AR(2) forecast is the more reliable one.

**Figure 3:**
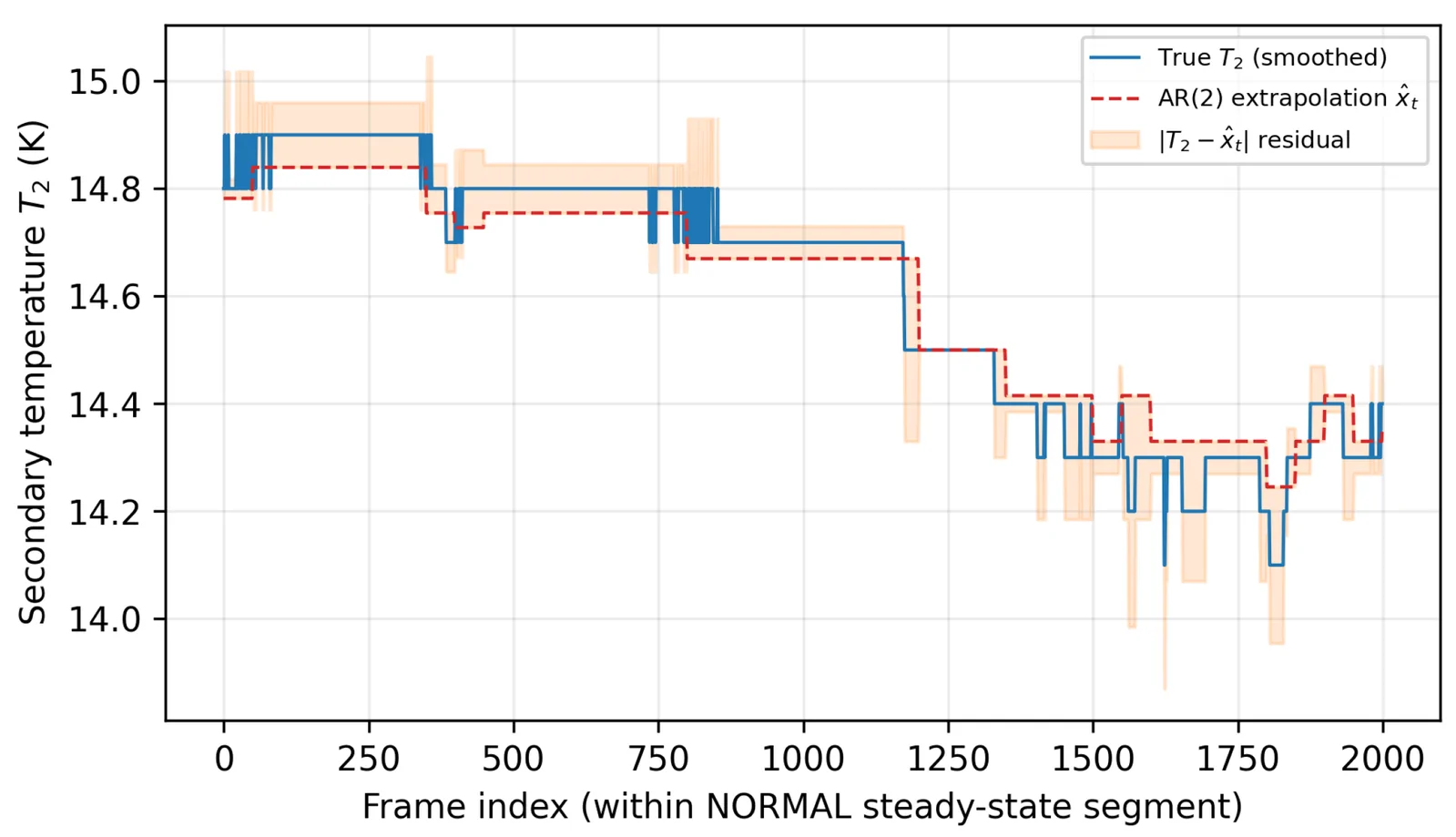
AR(2) one-step extrapolation on a real C-band NORMAL segment. The red dashed line is the predicted ${\hat{x}}_{t}$; the shaded band is the point-wise absolute residual. RMSE = 0.064 K.

Figure 3 verifies the one-step extrapolation accuracy of the fitted AR(2) model on a real C-band NORMAL steady-state segment. The predicted ${\hat{x}}_{t}$ (red dashed) tracks the true smoothed $T_{2}$ (blue) almost perfectly, with the shaded band showing the point-wise absolute residual $|T_{2}-{\hat{x}}_{t}|$; the segment RMSE is only 0.064 K, far below the orange threshold kσ used in Section 2.5.

A good one-step fit does not by itself license a 300-step forecast, so the extrapolation error is measured directly at several horizons rather than being inferred from the one-step residual. The evaluation uses a rolling origin: at each of a set of origins spaced 50 frames apart, the two most recent smoothed values seed the recursion of Equation (4), the model is rolled forward *h* steps, and the result is compared with the smoothed value actually observed *h* frames later. Only origins whose start and end both lie in the NORMAL steady state are kept. The AR(2) coefficients are those fitted on the pre-26 June 2024 segment, whereas the evaluation is run on the held-out segment after that date, so the fitting and evaluation segments are disjoint, and this is a held-out test rather than a refit. The same origins are used for a linear extrapolation of the same horizon, $x_{n+h}=x_{n}+\left(x_{n}-x_{n-1}\right)h$, which is the same-horizon reference introduced in Section 4.4. Table 2 reports the RMSE.

Note that RMSE is shown in kelvin between the *h*-step forecast and the smoothed $T_{2}$ actually observed *h* frames later; origins are spaced 50 frames apart and restricted to origins whose start and end both lie in the NORMAL steady state ($T_{2}<12.5$ K on L, <20 K on C and Q). Sample counts: 4577 (L), 100,392 (C) and 17,655 (Q). Bold marks the smaller error at each horizon.

Two findings should be stated plainly. First, at short horizons the linear extrapolation is the more accurate of the two: at h=1 its RMSE is 0.0031 K against 0.0254 K for AR(2) on L and 0.0129 K against 0.0248 K on C because an instantaneous difference estimates the slope over a single step well. Second, and more relevant here, the ordering reverses as the horizon grows: at h=300 the AR(2) RMSE is 0.1670, 0.8596 and 0.6321 K on L, C and Q, against 0.6929, 4.9919 and 2.4204 K for the linear rule, i.e., the linear error is 4.2, 5.8 and 3.8 times larger. The reason is structural: the AR(2) recursion has a stationary root ($a_{1}+a_{2}<1$), so its forecast is damped and its error saturates—on L, the RMSE is 0.1675 K at h=50 and still 0.1670 K at h=300—whereas the linear rule carries no such bound and its error grows with *h*. The horizon actually used for warning is H=300, and it is at that horizon, within this steady-state regime, that the AR(2) forecast is the more reliable of the two. This, rather than the one-step fit of Figure 3, is the empirical basis for treating “extrapolation breakthrough” as a precursor signal. AR(2) does not dominate at every horizon, and Section 4.4 shows that its advantage at H=300 translates into only a marginal difference in event-level warning metrics.

Table 2 is deliberately restricted to origins whose start and end both lie in the NORMAL steady state: that is the regime in which the AR(2) coefficients were fitted, in which the recursion of Equation (4) is well posed, and in which its stationary root damps the forecast. It is not, however, the regime in which warnings are generated. The orange precursor condition of Section 2.5 fires while the smoothed series is rising towards the bound, i.e., in the warming (RISE) regime; in the early segment, 74.9% of the orange frames on C and 89.8% on Q are raised while the classifier places the frame in the RISE state, the remainder falling in DROP or AMBIENT at the transition. Reporting only the steady-state error therefore risks overstating the accuracy at which the precursor test is evaluated. Table 3 closes that gap by repeating the same rolling-origin protocol with the origins instead restricted to frames that the four-state classifier places in the RISE (warming) modality; the seeds, the truth, the horizons, and the coefficients are unchanged.

Note that RMSE is shown in kelvin, computed exactly as in Table 2 but on origins whose four-state label is RISE; sample counts 6861 (L), 64,385 (C) and 18,302 (Q); same held-out segment and same pre-segment coefficients. For reference, the steady-state values of Table 2 at h=300 are 0.1670, 0.8596 and 0.6321 K on L, C and Q. Bold marks the smaller error at each horizon.

Two consequences follow. First, the warming regime error is an order of magnitude larger than the steady-state error that Table 2 reports: at h=300 the AR(2) RMSE rises from 0.1670 to 8.9631 K on L, from 0.8596 to 2.5636 K on C and from 0.6321 to 14.8135 K on Q. The steady-state figures must therefore not be read as the accuracy at which the precursor decision is taken, and the observation that the AR(2) error “saturates” holds only inside the fitted regime. The cause is the mean reversion of the recursion. Its stationary value is $c/(1-a_{1}-a_{2})$, about 12.2 K on L, 14.5 K on C and 15.9 K on Q. Once the temperature is genuinely rising above the normal bound, the forecast is pulled back towards the steady state and is biased low, whereas the unbounded linear rule continues the observed rise. Second, the ranking between the two rules is consequently not uniform in this regime: the linear rule is the more accurate of the two at h=1 on all three bands and remains so at h=300 on Q, while AR(2) is the more accurate at h=300 on L and C. Neither rule tracks the warming trajectory well.

This does not, however, translate into warning errors, and Section 4.4 gives the reason. The orange condition is a threshold-crossing test, so what decides the alarm is whether the trajectory crosses $T_{2}^{\mathrm{NORM}}+k\sigma$ while the trend is rising, not how accurately the endpoint 300 frames out is predicted. The horizon ablation shows directly that removing the look-ahead entirely leaves the event-level metrics unchanged. The role of the extrapolation is therefore to supply a crossing test rather than an accurate long-range temperature prediction, and the steady-state and warming regime errors of Table 2 and Table 3 bound that test from two sides instead of measuring the warning accuracy itself.

### 2.5. Three-Level Warning Finite State Machine

This module fuses the outputs of the first two modules (current state $s^{*}$, extrapolated value ${\hat{x}}_{n+H}$) and trend information into an executable warning level. The design principle is “graded progression, traceable reason”: the lower level hints at the trend, the higher level confirms the precursor or hard fault, and each level can explain to the operator “why the alarm”.

Identification factor and dead-zone. The “identification factor” is a quantity introduced in this paper to characterize “whether the temperature is undergoing a meaningful one-way trend”, used to identify whether the dewar temperature is stationary or continuously warming/cooling, thus serving as the direct basis for warning grading (yellow/orange). Specifically, the identification factor is defined as the window mean slope (trend rate), i.e., the temperature difference from the oldest frame to the newest frame in the window divided by the window length, giving a noise-robust linear trend slope:

(5) $$\mathrm{winSlope}=\frac{T_{2}^{\mathrm{newest}}-T_{2}^{\mathrm{oldest}}}{W}.$$

Compared with per-frame differencing, the window mean slope suppresses single-frame jitter. But under healthy steady state, thermal noise still makes the computed winSlope fluctuate slightly positive and negative, which are not real temperature trends. Therefore, a dead-zone δ is applied: when |winSlope|<δ, the temperature is judged stationary and the trend rate is recorded as 0, i.e., noise fluctuation is not treated as an effective trend. The dead-zone ensures that only when the identification factor truly crosses the threshold is a temperature trend acknowledged and possibly triggers a warning; otherwise, it is uniformly regarded as stationary, avoiding misreporting healthy steady-state thermal noise fluctuation as a low-level (yellow) warning.

Three-level decision. The three levels correspond to different severities, with a unified mechanism of “extrapolation breaks the steady-state upper bound + identification factor direction”:

- Red (RED, level = 3)—hard fault: captured by the FSM legality check. Physically, a transition must occur between NORMAL (steady state) and AMBIENT (ambient/vacuum loss); a direct jump between the two is an illegal hard jump, meaning an emergency stop fault such as sudden cooling loss. This level alarms immediately and is not suppressed by the consecutive-frame gate, to guarantee zero delay. The decision also requires that the previous state has been stably sustained for at least a certain number of frames, which prevents a brief misclassification during the system start-up preheating stage (when the temperature is not yet stable) from triggering a false red alarm.
- Orange (ORANGE, level = 2)—precursor warning: both “the extrapolation will break the steady-state upper bound” and “the temperature is still rising”, i.e.,
  (6) $${\hat{x}}_{n+H}>T_{2}^{\mathrm{norm}}+k\sigma \;\mathrm{and}\;\mathrm{winSlope}>\theta .$$
  Here, $T_{2}^{\mathrm{norm}}$ is the physical NORMAL upper bound of the band, and k=2 and σ is the AR(2) residual standard deviation, so the threshold is “physical NORMAL bound + 2 residual bands”. Two distinct quantities must not be confused here: $T_{2}^{\mathrm{norm}}$ is a fixed physical bound (12.5 K on L, 20.0 K on C and Q) and is the bound that actually enters the orange threshold of Equation (6), so that the threshold is $T_{2}^{\mathrm{norm}}+k\sigma$ and not $c_{0}+k\sigma$. The NORMAL cluster center $c_{0}$ (12.69, 16.32 and 17.82 K) is the state-conditioned sample mean used for classification and is not used in the threshold. The two differ by 0.19 K on L, which matters because the margin 2σ is itself only 0.24 K, so both are tabulated explicitly in Table 4 together with the resulting thresholds.
  The quantity θ is the minimum upward-trend threshold, i.e., the identification factor must reach this slope to be recognized as a meaningful warming trend. This equation means “following this inertia, the temperature will exceed the healthy band within the extrapolation horizon and is still climbing now”—that horizon being 5.17 h on L, 50 min on C and 2.83 h on Q —the most typical operable precursor.
- Yellow (YELLOW, level = 1)—early trend: the identification factor has reached the minimum trend threshold θ in magnitude, hinting that a temperature trend has appeared, but it has not yet reached the breakthrough magnitude:
  (7) $$\left|\mathrm{slopeEff}\right|\geq \theta ,\;\mathrm{slopeEff}=\left\{\begin{array}{cc} \mathrm{winSlope}, & |\mathrm{winSlope}|>\delta ,\\ 0, & |\mathrm{winSlope}|\leq \delta . \end{array}\right.$$

**Table 4:** Orange threshold components per band. $T_{2}^{\mathrm{norm}}$ is the physical NORMAL upper bound used in Equation (6); the NORMAL cluster center $c_{0}$ (Table 5) is shown alongside it because the two are easily confused.

Band	$T_{2}^{\mathrm{norm}}$ (K)	σ (K)	Orange Threshold (K)	NORMAL Center $c_{0}$ (K)
L	12.5	0.1193	12.5+2×0.1193=12.739	12.69
C	20.0	0.0715	20.0+2×0.0715=20.143	16.32
Q	20.0	0.0550	20.0+2×0.0550=20.110	17.82

**Table 5:** Data scale and statistical characteristics of the three bands.

Band	Raw	Bad-Point		Processed	$T_{2}$ Range After	NORMAL Cluster
	Frames	Frames	Rate	Frames	Filtering (K)	Center $c_{0}$ (K)
L	1,898,588	19,471	1.03%	1,898,539	0.50–99.90	12.69
C	14,171,620	567,358	4.00%	14,171,571	0.20–100.00	16.32
Q	4,324,261	1,622,033	37.51%	4,324,212	5.00–100.00	17.82

The level is triggered by θ, not by the dead-zone δ; δ enters only through the definition of slopeEff, as it does for orange. The level is therefore not triggered “once the dead-zone is crossed”: Equation (7) and the implementation both test against θ. Its role is to give early attention before orange, enabling the operator to troubleshoot in advance.

Parameter values, and how θ and δ relate. The deployed values are k=2, σ from the AR(2) fit (0.0715 K on C), θ=0.01 K/frame and δ=0.005 K/frame. Because winSlope is a per-frame slope over a window of W=50 frames, θ corresponds to a net $T_{2}$ change of θW=0.5 K across the window and δ to 0.25 K. Dividing by the median intervals of Table 6 gives $\theta \approx 1.6\times 10^{-4}$ K/s on L, $1.0\times 10^{-3}$ K/s on C and $2.9\times 10^{-4}$ K/s on Q, with δ half of these. Both are the program defaults of realtime_alert_full (–minrise, –deadzone) and are used unchanged in every experiment reported here.

Three points must be stated plainly. First, θ and δ are not among the parameters chosen by the ablation in Section 4.2: that ablation varies the report gate *N* only, and the statement that θ and δ were selected through it would be wrong. They are defaults inherited from the implementation, and we report them as such. Second, the two do not act independently: the dead-zone acts on slopeEff before the comparison against θ, and since the deployed values satisfy θ>δ, the dead-zone can never change the outcome—any |winSlope| that δ zeroes would in any case have failed |winSlope|≥θ. Re-running the C band with δ=0 reproduces the deployed result exactly (17,404 clusters, 3569 correct, precision 0.2051, F1 0.3403, recall 1.0000), so at this setting δ is inert rather than tuned. Third, δ is not inert in general: setting δ=0.02>θ makes the dead-zone the binding constraint, and precision rises to 0.4542 with F1 0.5794, but recall falls from 1.0000 to 0.8000. The interaction between the two is therefore real and material, and the present ablation does not explore it; we report this as an unexplored degree of freedom rather than as a tuned result and list it among the limitations in Section 6.

Consecutive-frame gate. Non-red warnings need to persist for ≥N frames before being issued (the ablation target of the report gate in Section 4.2, main N=3), to suppress single-frame glitch false alarms. Red is not suppressed. The key point is that this gate is applied at warning generation time by the program running parameter –report N, i.e., every warning appearing in the alarm log has passed the consecutive-frame check, strictly consistent with the subsequent offline evaluation protocol. The evaluation side does no simulation or recomputation, thus ensuring that the precision and recall reported in Section 4.1 (and throughout the paper) all come from truly generated warnings, not post-processing.

### 2.6. Online Processing Main Loop

The following main-loop pseudocode (per-frame online monitoring and three-level warning) summarizes the modules described in Section 2.1, Section 2.2, Section 2.3, Section 2.4 and Section 2.5, connected in a single data flow; each frame sequentially completes the five steps of “preprocessing → classification → extrapolation → grading → output”.

Pseudocode. Input: one raw sample $\left(t_{1},t_{2}\right)$ per frame; window W=50; horizon H=300; parameters k=2, σ, θ=0.01 K/frame, δ=0.005 K/frame; report gate *N*. *Output:* one alarm-log record $\left(t_{1},t_{2},L\right)$ per frame.

1. Preprocessing. If the sample is out of range, substitute it in place by the most recent valid value (Section 3.2): the frame advances the frame index but is not pushed onto the ring buffer of length *W*. A valid sample is pushed onto the ring buffer, and the smoothed observation $\left({\bar{T}}_{1},{\bar{T}}_{2}\right)$ together with the identification factor winSlope is computed from the valid samples in the window (Section 3.3).
2. Classification. Assign the nearest of the three non-AMBIENT cluster centers (NORMAL, RISE, DROP); AMBIENT is entered by the hard decision of Section 2.5, not by nearest-neighbor assignment. Keep the NORMAL center frozen and update the remaining centers incrementally (Section 2.3); test the resulting transition for FSM legality (Section 2.5).
3. Red (level 3). If the transition is a direct NORMAL↔AMBIENT jump—an illegal hard jump—and the previous state has been stably sustained for the required number of frames, set L=3 and go to step 6. Red alarms immediately and is not subject to the gate of step 5.
4. Extrapolation. Seed the AR(2) recursion of Equation (4) with the two most recent smoothed values and roll it forward *H* steps to obtain ${\hat{x}}_{n+H}$; apply the dead-zone to the identification factor, setting slopeEff=winSlope when |winSlope|>δ and slopeEff=0 otherwise (Section 2.4).
5. Grading and gate. If step 3 did not emit red, assign the level by
  (a) L=2 (orange) if ${\hat{x}}_{n+H}>T_{2}^{\mathrm{norm}}+k\sigma$ and winSlope>θ (Equation (6));
  (b) otherwise L=1 (yellow) if |slopeEff|≥θ (Equation (7));
  (c) otherwise L=0.
  Emit a non-red level only once it has been held for ≥N consecutive frames; red is exempt (Section 2.5).
6. Output. Append $\left(t_{1},t_{2},L\right)$ to the alarm log.

The key points of each line are explained below.

Per-frame flow. For each incoming monitoring value $\left(t_{1},t_{2}\right)$, the main loop sequentially executes the following five steps. First, bad-point filtering handles out-of-range samples: the frame is substituted in place by the most recent valid value and is skipped by the window, corresponding to the criterion in Section 3.2. Second, a valid frame is pushed into the ring buffer of length *W*, and the smoothed observation $\left({\bar{T}}_{1},{\bar{T}}_{2}\right)$ and the identification factor winSlope are computed (corresponding to Section 3.3). Third, online k-means four-state classification is performed (nearest-neighbor assignment + incremental update, NORMAL center frozen, corresponding to Section 2.3), and the FSM legality check captures the red-level hard jump (land immediately, not suppressed by the consecutive-frame gate, corresponding to Section 2.5). Fourth, the AR(2) extrapolation is performed, and the identification factor winSlope is processed through the dead-zone δ to obtain the effective slope (corresponding to Section 2.4). Fifth, for frames that did not trigger red, orange/yellow/no warning (level 0) is given according to “extrapolation breakthrough + identification factor direction”, and the consecutive *N*-frame gate is applied to non-red levels (corresponding to Section 2.5 and the report-gate ablation in Section 4.2), writing $\left(t_{1},t_{2},L\right)$ to the alarm log.

Why lightweight and real-time. Every step in the loop is a fixed-complexity per-frame operation whose cost does not grow with the amount of history already seen. Bad-point substitution is an O(1) table lookup, smoothing is an O(1) ring-buffer mean, classification is a four-state nearest-neighbor assignment with an O(1) incremental update, and the FSM is an O(1) state comparison. There is no training phase, no batch processing and no full-history rescan. The AR(2) step is the one exception, and it must be stated precisely. The recursion itself advances the state by a single frame at O(1) per step (Equation (4)), but obtaining the forecast ${\hat{x}}_{n+H}$ used by the orange decision requires iterating it *H* times, so this step is O(H), and it is this step that determines the per-frame cost. The per-frame complexity of the loop is therefore O(H) and not O(1). It is constant with respect to the length of the dataset but not with respect to the forecast horizon, and the earlier claim that the whole loop is O(1) conflated the two. With the horizon fixed at its deployed value of H=300 frames, the cost is a constant in practice, which is what the measured per-frame time in Section 4.5 reflects. The O(1) claim held only under that fixed horizon, and the dependence on *H* is now stated explicitly. This also echoes the statement that “classification and extrapolation are parallel but decoupled” in Section 2.1—the two are computed independently within the same frame and only jointly drive the FSM at the summary step of this loop without blocking each other.

## 3. Data Acquisition and Preprocessing

### 3.1. Data Source and Scale

The data used as the case study come from the L/C/Q band cryogenic dewar systems of a 26 m radio telescope, whose on-board sensors continuously record the two-stage temperatures and the dewar vacuum. The real dewar monitoring data are comma-separated CSV files containing r_date, r_time, tempStage1, tempStage2 (the L band additionally contains a vacuum column). Taking 26 June 2024 as the dividing date, the segment before this date comes from the authors’ published prior work [30] and is used for coefficient fitting and ablation experiments; the segment after is used for the main evaluation. The sampling intervals of the three bands are inconsistent. Measured directly on the record, the median interval is about 62 s for L in the later segment, while the early segment is genuinely bimodal at about 32 s and 62 s (44.3% and 52.1% of frames). It is about 10 s for C in both segments and about 34 s for Q in both segments. The 3–4 s and 67–68 s spacings that appear in the Q band are initialization artifacts confined to the first days of the archive in October 2021 and account for about 0.12% of frames; they are not a second sampling mode, and the earlier claim of a Q-band bimodal pattern is withdrawn. To eliminate the band dependence introduced by time conversion, this paper uniformly uses the frame index as the unit of window and horizon. Consequently, the same frame count denotes different physical durations on different bands, so Table 6 gives the conversion explicitly and the lead time is reported per band wherever a duration is quoted; no single figure such as “about 5.2 h” applies to all three bands. Table 5 gives the raw data scale, bad-point rate, and temperature range of each band (a bad point means a record where $T_{1}\notin \left[0,400\right]$ K or $T_{2}\notin \left[0,100\right]$ K is out of range, such as a sensor anomaly value −3 K or 50,319.8 K).

Note that the raw frame count is the total number of CSV rows. A bad-point frame is a row failing the criterion above ($T_{1}\notin \left[0,400\right]$ K or $T_{2}\notin \left[0,100\right]$ K). As described in Section 3.2, such a sample is not deleted—it is substituted in place by the most recent valid observation, so that the frame index and the time base are preserved, while the substituted value does not enter the sliding window. This is why the bad-point rate has no effect on the frame count. The processed-frame count is the number of frames that enter the evaluation; it is the raw count minus the 49 warm-up frames consumed by the sliding window of W=50, not minus the bad points, and it is therefore the same kind of constant offset on every band. The offset is 49 frames and not 48 since the sliding window of W=50 consumes the first 49 frames as warm-up. The processed-frame column is therefore obtained by subtracting this warm-up offset, and it does not represent the removal of malformed (bad-point) records—malformed rows are substituted rather than deleted and do not reduce the frame count. The $T_{2}$ range is the range of the in-range (valid) readings and does not contain the −3 K artifact; it covers the whole record, including refrigeration stoppages and vacuum-loss episodes. The NORMAL center is the $T_{2}$ centroid of the NORMAL cluster (Section 2.3), denoted $c_{0}$; it is used for classification only and is not the $T_{2}^{\mathrm{norm}}$ appearing in the orange threshold of Equation (6)—the two differ by 0.19 K on L and are tabulated side by side in Table 4. Steady-state operation fluctuates in a band far narrower than the full-record range—about 11.9–13.0 K on L—whereas the $T_{2}>20$ K episodes discussed in Section 4.7 are vacuum-loss periods and lie outside the steady state, so the two statements do not conflict. The Q band’s bad-point rate, which is as high as 37.5%, highlights the necessity of bad-point filtering.

Because all windows and horizons in this paper are counted in frames, the same frame count corresponds to different durations per band. Table 6 makes this conversion explicit, so that every duration quoted later in the paper can be read off per band rather than being approximated by a single figure.

Note that the interval is the median of the per-frame spacing measured on the processed record. For the L band, the early segment contains two comparably frequent spacings, so a 300-frame window covers a range rather than a single duration; the later segment is single-moded at 62 s. The main evaluation is run on the later segment, where the relevant-zone window relWin = 300 and the extrapolation horizon *H* = 300 correspond to 5.17 h on L, 50 min on C and 2.83 h on Q.

### 3.2. Bad-Point Filtering

The raw CSV is persisted to disk by the field acquisition system at fixed intervals. Owing to transient sensor disconnection, communication frame misalignment, or write exceptions, wild values that clearly exceed the physical range appear. Taking the dewar temperature as an example, both the primary temperature $T_{1}$ and the secondary temperature $T_{2}$ have physically reachable upper bounds. From device specifications and historical observations, this paper adopts the reasonable range $T_{1}\in \left[0,400\right]\;K$ and $T_{2}\in \left[0,100\right]\;K$ (under normal operation $T_{2}$ is about 12–21 K, see Table 5). Any channel outside this range (such as a sensor anomaly value −3 K or 50,319.8 K) is judged as a bad point.

Bad-point handling adopts the most conservative substitution strategy: the point is replaced in place by the most recent valid observation, and that frame is not written into the subsequent sliding window. This design has three considerations. First, substituting rather than zeroing or interpolating avoids introducing spurious steps or slopes under a high-noise background, thus protecting the mean and the online k-means cluster centers from contamination (see Section 2.3). Second, not entering the window means the bad point participates neither in the current smoothing nor in the extrapolation, ensuring that the downstream warning is built entirely on credible samples. Third, this step only performs data cleaning and does not physically rewrite or simulate the temperature series, so it does not affect the authenticity and interpretability of the warning mechanism in Section 2.5.

Terminology. Three words appear around this rule, and each has exactly one meaning in this paper. A bad point is a frame whose $T_{1}$ or $T_{2}$ falls outside the accepted range above. Substitution, also written replacement in place, is what happens to such a frame: its two channels are overwritten by the most recent valid observation, while the frame itself stays in the series with its frame index and timestamp unchanged. A substituted frame is skipped by the sliding window, i.e., its value is not enqueued in the ring buffer and enters neither the smoothed mean, nor the online k-means center update, nor the AR(2) recursion. Consequently, the phrase “bad points are removed” is not used in this paper and is not to be read as a description of this rule: no row is ever deleted, and no substituted value ever enters a window. The phrase “the series keeps its continuity” means only that the frame index and the time base are preserved, so that the frame counts on which the window *W*, the horizon *H* and the lead-time table are based stay valid.

The two corrections that might be considered instead are both unsafe here, and the reason can be made quantitative. Clipping an out-of-range reading to a boundary such as 0 K does not restore a plausible value: under normal operation $T_{2}\approx 12$–21 K, so clipping replaces a true reading by one 12–21 K away from it. The consequence follows from the decision margins of Section 2.5, where the identification factor is the mean slope over the W=50 frame window and the orange decision requires that slope to exceed θ=0.01 K/frame. A single clipped sample therefore contributes about 12/50 to 21/50, i.e., 0.24 to 0.42 K/frame, some 24 to 42 times the threshold. Because the AR(2) recursion is second order, the same step enters the innovation at the same time, so both orange conditions would be met, and a harmless sensor glitch would be turned into a spurious orange or even red warning. Deleting the frame is unsuitable for a structural reason rather than a statistical one. The window *W*, the horizon *H*, the relevant-zone windows relWin and postWin and the consecutive-frame gate *N* are all counted in frame indices, so removing rows silently shortens the effective horizon and distorts the lead time reported in Table 6. Substitution avoids both—the frame count, and hence the time base, is preserved, while the invalid value itself never enters the smoothing, the k-means center update, or the AR(2) recursion.

Interpolating from the two nearest valid neighbors is not obviously safer on this data either. Bad points are not isolated. On the Q band, they account for 37.5% of frames and often occur in runs, so the two nearest valid neighbors of a bad frame may lie far apart. A straight line between them then imputes a sustained non-zero slope across the whole run—which is exactly the quantity that the identification factor then reads and that the orange decision thresholds at θ. Substitution instead holds the last valid value, giving a slope that is zero within the run and therefore below θ, so it cannot by itself escalate the warning level. This remains the one comparison we would regard as worth running as an ablation, and we report it as not done. The full per-band records are not held with the manuscript and are re-exported from the acquisition database, so we cannot give the C and Q precision, F1 and out-of-zone warning-frame fraction under interpolation, and we do not state a result we have not measured.

It must be emphasized that the necessity of bad-point filtering is determined by the data itself: as shown in Table 5, the Q band bad-point rate reaches 37.5%; if the raw series directly drives classification and extrapolation, the cluster centers and AR(2) coefficients would be severely biased. Precisely because of this, the evaluation frame counts of all experiments in this paper are counted under the “after bad points are substituted” protocol (see the note of Table 5), ensuring that the conclusions rest on cleaned real data.

High-temperature faults and the 100 K bound. The two bounds are deliberately asymmetric, and the asymmetry answers the natural objection that a rule which rejects $T_{2}>100$ K must also reject the faults that the method is meant to catch. The $T_{1}$ bound of 400 K lies far above the highest temperature a warm-up can physically produce: a dewar returning to room temperature reads about 300 K on the first stage, so the first-stage channel is never filtered during a real fault. The $T_{2}$ bound of 100 K, in contrast, is approached only when the second stage comes close to ambient because $T_{2}$ stays at 12–21 K during normal operation (Table 5). A high-temperature fault is therefore declared on the first-stage channel and with a wide margin. The AMBIENT state is entered when ${\bar{T}}_{1}$ exceeds the ambient threshold of the band (284 K on C and Q, 293 K on L), which is the AMBIENT hard decision of Section 2.5 and is precisely the physical statement that the dewar has come back to room temperature, i.e., the condition that defines the 290 K episode discussed in Section 2.3. In the real record, the alarm is raised while the second stage is still far below its bound: the red frames of the C band on 25 February 2024, 24 August 2024 and 27 May 2026 carry ${\bar{T}}_{1}=290.5$, 300.7 and 294.4 K together with ${\bar{T}}_{2}=29.2$, 30.7 and 29.8 K, i.e., roughly 70 K of margin below the 100 K bound at the moment that the fault is declared. Frames with $T_{2}>100$ K do occur in this record and are substituted. The largest such excursion, the vacuum-loss episode in which the second stage reaches 156.0 K (Section 4.7), lies inside a fault that has already been reported, and substitution preserves the frame index, so the alarm time is unaffected.

Sensitivity to the upper $T_{2}$ bound. It can be shown directly that the bound is a cleaning rule rather than an alarm criterion. The whole three-band record was rerun with the upper $T_{2}$ bound raised from 100 K to 400 K, which makes it inactive on this data (the bound is a compile-time constant of the implementation, so the comparison is exact). No detection instant moves. The first yellow frame occurs at the same timestamp in both settings on all three bands (L 22 March 2023 10:07:51, C 15 October 2021 16:53:26, Q 7 October 2021 16:14:17), the first orange frame is unchanged where it exists (C 24 October 2021 15:20:33, Q 5 November 2021 04:31:06), and on the held-out segment the precursor recall is identical (C 267/267, Q 616/616, both 1.0000). No real fault is therefore lost through this bound. What the bound does change is the volume and the label of the warnings. Flagged frames increase (held-out segment: C from 281,775 to 304,293, Q from 221,456 to 251,997, L from 49,093 to 52,694) and precision falls correspondingly (C 0.1373 to 0.1294, Q 0.2391 to 0.1852) because the extra high-temperature frames are no longer discarded but enter the window as valid observations that carry a trend of their own. The red frames of the C band also disappear, and the same warm-up is reported at orange level (hard fault recall 1.0000 to 0.0000). The reason is that the red level is reserved for a transition that the finite-state machine registers as a direct NORMAL-to-AMBIENT step (Section 2.5). With the bound, the substituted $T_{2}$ plateau holds the state at NORMAL up to the frame that issues the first warning, and the transition is flagged when it happens. Without it, the state at that same frame is already AMBIENT, so no such step is registered, and the escalation is not triggered. The bound thus acts conservatively in both directions: it discards readings that a second stage cannot physically produce, which raises precision, and where it changes a warning level it escalates the label rather than relaxing it. All results reported in this paper use the deployed value of 100 K.

### 3.3. Sliding-Window Smoothing

After bad-point substitution, a single-frame observation still carries sampling noise and small jitter. To suppress high-frequency noise and highlight the trend-like slow variation of temperature (which is the main carrier of fault precursors), this paper applies a fixed-length sliding-window mean smoothing to each frame. Let the window length be W=50 frames (frames are used as the unit instead of time, for the reason discussed in Section 3.1 on inconsistent sampling intervals). Let $w_{1}\left[i\right]$ and $w_{2}\left[i\right]$ be the primary/secondary temperatures of the *i*-th frame in the window. The smoothed observation of this frame is

(8) $${\bar{T}}_{1}=\frac{1}{W}\sum_{i=0}^{W-1}w_{1}\left[i\right],\;{\bar{T}}_{2}=\frac{1}{W}\sum_{i=0}^{W-1}w_{2}\left[i\right].$$

The window is implemented as a ring buffer, with only one enqueue and one dequeue per frame, so the time complexity is O(1), suitable for real-time embedded deployment (per-frame overhead in Section 4.5).

The smoothed ${\bar{T}}_{1},{\bar{T}}_{2}$ of Equation (8) are the unified starting point of all subsequent modules. On one hand, they are the input features of online k-means four-state classification (Equation (1)); on the other hand, ${\bar{T}}_{2}$ enters the AR(2) extrapolation (Equations (3) and (4)) and the identification factor (window mean slope, Equation (5)). The window length *W* creates a trade-off between network noise and trend sensitivity: a window that is too small creates insufficient smoothing and easily triggers noise-level false alarms, while a window that is too lags the fault precursor detection. This paper uses W=50 frames as the implementation default (Section 2.5); the ablation of Section 4.2 varies only the report gate *N* and does not select *W*.

## 4. Experiments and Results

Evaluation protocol. A warning event cluster is defined as a continuous level ≥ 1 segment; the real anomaly events used as the evaluation baseline are extracted from the continuous warning segments in the alarm log (see Section 5 for data characteristic notes). Let a real anomaly event occupy the frame interval [start,end]. Its relevant zone is the union of three windows, written explicitly as

$$\begin{array}{cc} R & =[\;start-relWin,\;start\;]\;\cup \;[\;start,\;end\;]\;\cup \;[\;end,\;end+postWin\;]\\ \; & =[\;start-relWin,\;end+postWin\;], \end{array}$$

namely the pre-warning window [start−relWin,start] of relWin frames before the event (mainly 300, covering the detectable precursor), the event itself [start,end], and the post-event window [end,end+postWin] of postWin frames after it (50, covering continued observation). The interval is clipped to the record bounds. The event frames themselves lie inside the zone, so *R* is one contiguous interval and not two disjoint ones: the pre-event window and the event interval are joined by the event rather than separated from it. Recall is the proportion of events containing ≥1 warning frame within *R*; precision is the proportion of warning clusters whose center falls in *R*; F1 is the harmonic mean; out-of-zone warning-frame fraction (OWF) is the proportion of warning frames that fall outside *R*; the pre-warning window recall is the hit rate within the pre-warning window [start−relWin,start] alone. OWF is deliberately not called a false positive rate; it is the complement of a precision-like quantity taken over warned frames only, whereas the standard false positive rate is FP/(FP + TN) taken over all negatives. Here, TN is dominated by the millions of unwarned out-of-zone frames, so the standard rate is of order $10^{-3}$ and does not discriminate between settings. The quantity is therefore reported as the out-of-zone warning-frame fraction (OWF) and not as a false positive rate.

Data segmentation note. The record of each band is divided by the date 26 June 2024. The early segment (before the date, from the literature [30]) is used for coefficient fitting, ablation, window sensitivity and baseline control experiments; the held-out segment (strictly after the date) is used for the main evaluation of Table 7. No frame of the held-out segment enters the offline fitting of any parameter: the AR(2) coefficients, the four-state thresholds and the initial four-state centers are fitted on the early segment only. The evaluation run itself is nevertheless executed over the whole record in its natural causal order—exactly as the deployed program does—and only the frames and the real anomaly events after the dividing date are scored. This keeps the warm-up of the online state (sliding window, drifting cluster centers) that any deployment would have, while keeping every reported metric free of data used for fitting. Unless otherwise specified, the main evaluation uses the held-out segment, and the other control experiments use the early segment.

### 4.1. Main Evaluation (Held-Out Segment, Event-Level, relWin = 300)

The held-out segment contains, after bad-point filtering, 1,066,126 L frames, 6,345,157 C frames and 1,951,578 Q frames, and it contains 0, 267 and 616 real anomaly events, respectively. These event counts are exactly the full-record events that fall after the dividing date (C: 342−75=267; Q: 1364−748=616), i.e., no event of the calibration segment enters the main evaluation. The L band has no real anomaly event in the held-out segment. Every metric in this table is scored against that event set, so no event-level figure can be formed for L. Its recall, precision and F1 would all have an empty denominator, and its out-of-zone warning-frame fraction has no relevant zone to refer to. The row is therefore marked as “–”, meaning undefined, rather than 0, which would wrongly suggest a measured result of zero. Because this event list is extracted from the alarm log of this pipeline, the precision and F1 columns of this table measure agreement with this method’s own event list rather than independent fault detection accuracy, as stated in the scope paragraph below and in Section 5. This also indicates that the algorithm produced no red (hard fault) false alarm during the monitoring period after the dividing date, verifying that the FSM’s zero-delay capture of emergency stop hard jumps in Section 2.5 does not produce false red alarms, and the steady-state safety is good. The C and Q bands achieve event-level recall of 1.0000 at both N=1 and N=3, i.e., 100% of the held-out events have at least one warning frame hit within the relevant zone. The C band’s hard fault recall is 1.0000(1/1); its single held-out red event (25 August 2024) was captured. The second red event of the full record (25 February 2024) lies in the calibration segment and is therefore excluded. The pre-warning window recalls are 0.9663 (C, N=1), 0.9513 (C, N=3), 0.9968 (Q, N=1) and 0.9935 (Q, N=3), showing that the vast majority of faults already exhibited detectable precursors within the relevant zone before their start, i.e., within 50 min on C and 2.83 h on Q (relWin = 300 frames converted per band according to Table 6; the L band has no event in the held-out segment), and the pre-warning capability is established.

Which data and which *N* produce Table 7. Every number in Table 7 is computed on the held-out segment only (frames strictly after 26 June 2024), with relWin = 300, postWin = 50 and minLvl = 1; the offline-fitted parameters come from the early segment alone. Both N=1 (no consecutive-frame gate, so that every warning frame is reported) and N=3 (the setting recommended by the ablation of Section 4.2) are reported, so that the value behind each figure is explicit. The two settings flag almost the same frames—the OWF differs by less than 0.001 (C 0.8828 vs. 0.8829; Q 0.7067 vs. 0.7069)—while the number of warning frames drops by about two thirds at N=3 (C 281,775→93,928; Q 221,456→73,818). The consecutive-frame gate therefore mainly changes the granularity at which warnings are grouped into clusters and the volume of warnings an operator receives, rather than which frames are regarded as anomalous. On the held-out segment this granularity change is band-dependent: precision improves for Q (0.2391→0.2906, F1 0.3860→0.4504) and decreases slightly for C (0.1373→0.1146, F1 0.2415→0.2056). We retain N=3 as the recommended setting for the reasons given in Section 4.2 and report both values here, without claiming that N=3 dominates N=1 on every band.

It should be noted that the precision in Table 7 (C 0.1373/0.1146, Q 0.2391/0.2906) and the out-of-zone warning-frame fraction (C 0.8828/0.8829, Q 0.7067/0.7069) seem low. This is not a method defect but is caused by the high sparsity of real faults in year-round monitoring—warning clusters mostly fall outside the relevant zone of faults. Since this baseline event is extracted from the continuous segments of the alarm log and therefore shares its source with the warnings (Section 5). The upward bias of the recall-type metrics is a structural deviation, so recall cannot be used as strict evidence of “independent fault capture rate”. This paper pays more attention to the absolute value of the precision and the relative improvement over the baseline below (Section 4.4), which is the substantive embodiment of the method’s validity.

Scope of the precision and F1 reported in this paper (agreement with the method’s own event list, not independent detection accuracy). The precision and F1 values of Table 7, Table 8, Table 9 and Table 10, and the values quoted from them in the abstract, measure the agreement between a method’s warning frames and the relevant zones of the event list defined in this paper. They are not an independent fault detection accuracy. The event list is itself extracted from the continuous warning segments of the alarm log of this very pipeline, and the consequence is two-sided. On one side, a warning frame of any method—this method included—that falls on a physically real degradation episode which the alarm log does not turn into an event is scored as a false alarm, so part of the false alarm volume of every row may be real but unlabeled faults. On the other side, each sustained warning cluster of this method automatically generates the relevant zone in which its own frames are counted as true positives, whereas an equally sustained cluster of a competing method is credited only if it happens to overlap with one of ours. The absolute values are therefore optimistic for the method that defines the events. Consequently, the absolute precision and F1 are reported only as a fixed-protocol agreement indicator, and the substantive evidence is the relative comparison: every row of Table 10 is scored by exactly the same criterion on the same frames and against the same event list (Section 4.4) so that the order-of-magnitude gap is a like-for-like result. The physical cross-check of the raw sensor record reported later in this section is the partial substitute for independent ground truth that this installation allows.

How a warning segment becomes a “real anomaly event”. Because the event set is derived from the same alarm log that carries the warnings, the extraction rule is stated here explicitly so that the reader can judge what the set does and does not contain. A candidate event is a maximal run of consecutive frames carrying warning level ≥2, i.e., orange or red. Yellow warnings (level = 1) are not counted, which is the first and coarsest rejection of noise. Two further de-noising conditions are applied: adjacent runs separated by at most 50 frames are merged into a single event, so that one physical fault interrupted by brief dips is not counted several times. Any run shorter than 5 frames is discarded so that a single-frame glitch cannot become an event at all. An event containing at least one red frame is labeled RED, otherwise ORANGE. Both thresholds are fixed constants of the labeling procedure and are not tuned per band or per experiment.

Independent physical cross-check of the labeled events. The rule above is same-source with the warnings by construction, so it can establish that a labeled event is a sustained warning but not that it is an independent fault. We therefore cross-checked a subset of the labels against the raw sensor record, which is independent of the alarm log, using the physical shutdown criterion of the dewar. A genuine refrigeration stoppage drives the first-stage temperature back into the ambient range, above 284 K for the C and Q bands and above 293 K for L, whereas the normal range is below 54 K for the first stage and below 20 K for the second. The C band contains two RED events in the whole record, and both fall on days on which the raw record does show an excursion into the ambient range. On 25 February 2024 the first-stage temperature peaked at 291.2 K, with 3767 of 8480 frames (44%) above 284 K, and on 25 August 2024 it peaked at 302.8 K, with 3855 of 8491 frames (45%) above 284 K. Both RED events thus correspond to real refrigeration stoppages rather than to noise. For the ORANGE class we checked the first five events of the held-out C-band segment (12 July 2024, 6 August 2024, and three on 8 August 2024). In every one of them, the raw record lies outside the normal range, with first-stage peaks of 67.3–110.5 K against the 54 K limit and second-stage peaks of 23.3–27.2 K against the 20 K limit. The limits of this check should be stated plainly. It confirms that the labeled events are physically real, but it cannot confirm that the label set is complete, and with only two RED events in the entire record it does not establish the hard fault miss rate. Formally maintained maintenance or shutdown logs were not available for this installation; the ambient-temperature criterion used above is the closest physical substitute, and obtaining such logs remains necessary, as noted in Section 5.

### 4.2. Report-Gate Ablation (Early Segment, Real–Report N)

For each *N*, the program running parameter –report N genuinely applies the consecutive-frame gate at warning generation time, and its ablation effect is evaluated; the results are summarized in Table 8:

**Table 8:** Report-gate ablation results (early segment, relWin = 300, real anomaly events: C = 75, Q = 748, L = 0).

Band	Precision				F1			
	N=1	N=3	N=5	N=10	N=1	N=3	N=5	N=10
L	–	–	–	–	–	–	–	–
C	0.0636	0.2051	0.2060	0.2065	0.1196	0.3403	0.3417	0.3424
Q	0.2068	0.2484	0.2495	0.2500	0.3427	0.3979	0.3994	0.4000

Result interpretation (for the reader). To make this section accessible, the “consecutive-frame gate” can be stated in one sentence: it acts like a debounce switch—a non-red warning must appear continuously for at least *N* frames before it is formally issued. If it only flickers for a single frame and then returns to normal, it is treated as a glitch and not reported. Hence the larger *N* is, the less sensitive the system is to single-frame noise, but at the cost of the warning being delayed by “*N* frames”.

Why recall is always 1.0000 (to be treated with caution). The recall of all three bands in Table 8 is 1.0000, i.e., all real anomaly events in the early segment (C = 75, Q = 748, see the header of Table 8) have at least one warning frame hit within the relevant zone, seemingly a “zero miss”. But this high value is a structural bias, not evidence that the independent miss rate is zero. The “real anomaly events” of this experiment are themselves extracted from the continuous warning segments of the same alarm log (same-source with the warnings, see the note at the end of Section 4.1), meaning the warnings are used as their own reference answer, so recall is naturally close to perfect. Therefore, this section does not take recall as evidence of method validity but places the conclusion on precision and F1—they measure “how many of the issued warnings are real”.

How debouncing improves precision. Setting N=1 is equivalent to turning off the debounce switch: any single-frame anomaly alarms immediately, so a large number of transient glitches are falsely reported, and precision is very low (C only 0.0636, Q only 0.2068, with F1 only 0.1196, 0.3427). Raising *N* to 3 requires three consecutive frames before alarming; most single-frame glitches are filtered out and precision more than triples (C rises to 0.2051, Q to 0.2484; F1 rises to 0.3403, 0.3979).

Why *N* need not be larger. When *N* is further increased to 5 or 10, the glitches are already filtered out, and precision and F1 only increase marginally (C precision 0.2060/0.2065, F1 0.3417/0.3424; Q also only rises slowly), with the gain clearly tapering off. In other words, the “dividend” of debouncing is almost entirely concentrated in the single step from N=1 to N=3; increasing *N* further only linearly lengthens the warning delay without buying much additional false alarm reduction.

Conclusion. Balancing “false alarm reduction” against “warning-delay cost”, N=3 is the most cost-effective main report gate. It lowers false warnings caused by single-frame jitter by about 3 times yet incurs no loss in capturing real precursors (recall unchanged). The warning delay is only 3 frames (30 s on C at 10 s/frame and about 1.7 min on Q at 34 s/frame; the L band has no event in this test), which is fully acceptable in engineering.

### 4.3. Relevant-Zone Window Sensitivity (Early Segment, Report = 3)

Change the relevant-zone forward frames relWin and observe the trade-off between precursor capture and false alarms; the results are summarized in Table 9:

**Table 9:** Relevant-zone window sensitivity results (early segment, report = 3).

Band	Precision			OWF		
	relWin = 200	relWin = 300	relWin = 500	relWin = 200	relWin = 300	relWin = 500
C	0.2014	0.2051	0.2146	0.7970	0.7933	0.7838
Q	0.2375	0.2484	0.2651	0.7600	0.7492	0.7325

Increasing relWin expands the relevant zone forward wider, and more warning frames are “wrapped into” the relevant zone, so precision rises slightly and the out-of-zone warning-frame fraction (OWF) drops slightly, as expected. Specific values: the C band precision rises from 0.2014 at relWin = 200 to 0.2051 (relWin = 300) to 0.2146 (relWin = 500), the OWF drops from 0.7970 to 0.7933 to 0.7838; the Q band precision rises from 0.2375 to 0.2484 to 0.2651, the OWF drops from 0.7600 to 0.7492 to 0.7325, with a consistent trend. Converted per band (10 s/frame on C, 34 s/frame on Q), relWin = 200/300/500 correspond to pre-warning windows of about 33/50/83 min on C and about 1.9/2.8/4.7 h on Q. The L band has no event in this test. It should be noted that relWin only changes the width of the relevant zone (evaluation protocol) and does not affect the generation of the warning itself, so recall and pre-warning window recall change passively with the window. This section focuses on its impact on precision and OWF and does not list recall separately. Overall, relWin = 300 frames—i.e., 50 min on C and 2.83 h on Q, the two bands that carry events in this ablation—achieves a balance between covering a reasonable pre-warning window and avoiding excessive loosening of the protocol, and the increment has clearly slowed down by 300→500. Therefore, relWin = 300 is chosen as the unified main setting of the whole paper (together with the main evaluation in Section 4.1 and N=3 in Section 4.2, forming the main configuration relWin = 300, postWin = 50, N=3; all tables in this paper are computed under this protocol with minLvl = 1).

### 4.4. Baseline Method Comparison (Calibration (Early) Segment, Report = 3)

**Figure 4:**
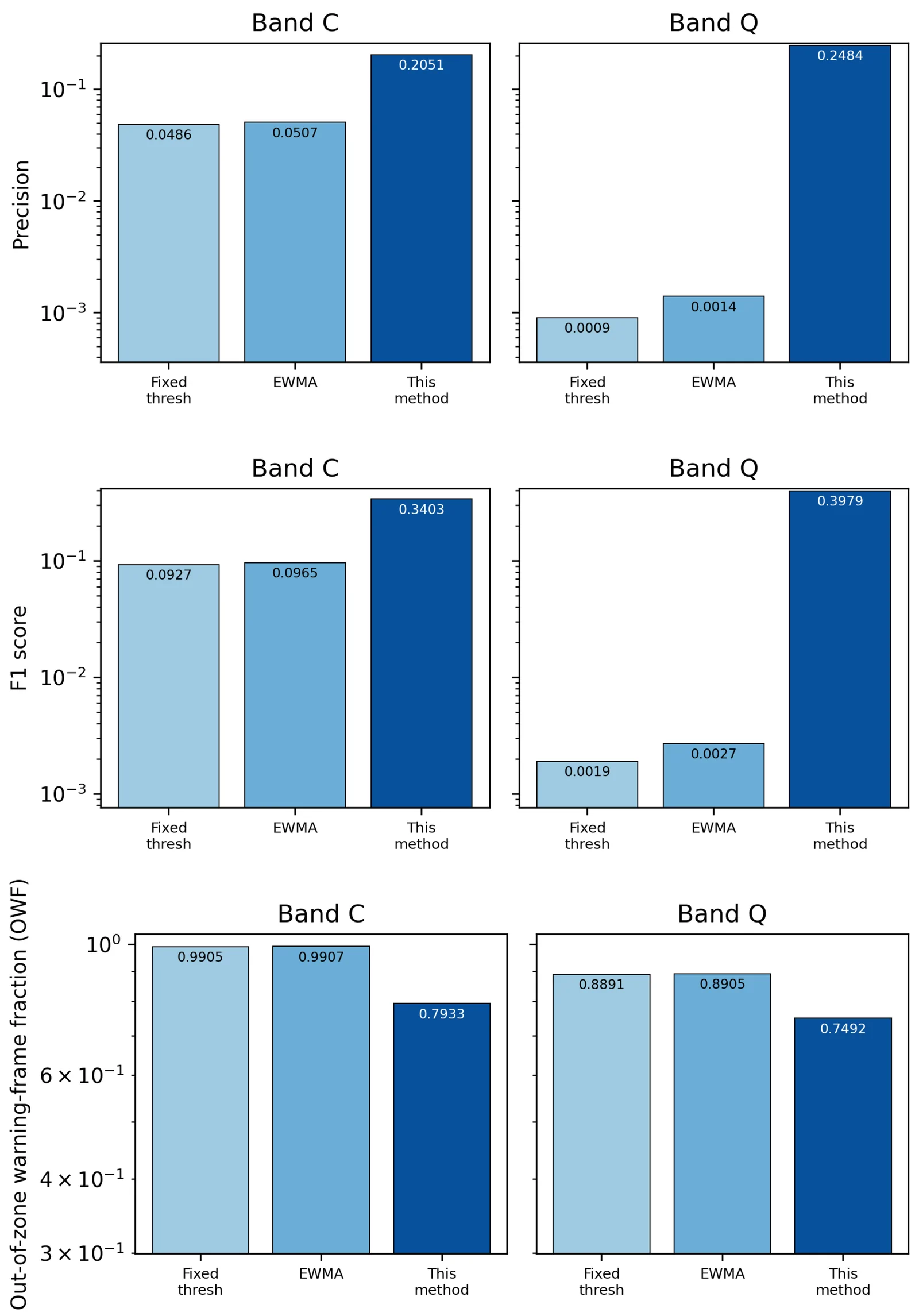
Baseline comparison on the calibration (early) segment (report = 3, relWin = 300, not a held-out evaluation) in log scale. Top: precision; middle: F1; bottom: out-of-zone warning-frame fraction. For each metric the three methods are Fixed threshold, EWMA, and This method; the C/Q panels share the same vertical axis. The L band is not plotted because it has no real anomaly event in this segment: its precision and F1 are identically zero for every method and its out-of-zone warning-frame fraction is undefined, so a bar chart would carry no comparison; the corresponding L values are given in Table 10.

Provenance of the numbers in this section (calibration segment, not a held-out test). Table 10, Figure 4 and the baseline numbers quoted in the abstract are computed on the calibration (early) segment—the frames before the dividing date 26 June 2024—and are scored against the event list of that same segment. That segment is the one from which the offline parameters of this method are fitted (the AR(2) coefficients, the four-state thresholds and the initial four-state centers, Section 4.1), so it is not an independent test set, and the absolute precision and F1 values of Table 10 are not held-out performance. The held-out evaluation of this method—frames strictly after the dividing date, with no held-out frame entering any offline fit—is reported in Table 7. We have kept the baseline comparison in this section rather than move it to the held-out segment. The two baselines are a fixed absolute-temperature threshold and a one-frame EWMA extrapolation, which carry no fitted parameters at all. What the comparison establishes is the relative ordering of methods under one and the same scoring protocol, and the gap reported below is claimed only as such a relative result, not as held-out evidence. The control experiments of Section 4.2, Section 4.3 and Section 4.4 share this segment so that the whole ablation series is read on one and the same data.

Three controls are compared on the real report = 3 products. The fixed-threshold method (baseline_threshold) only alarms on absolute temperature crossing, with no trend extrapolation and no state adaptation. The EWMA trend-extrapolation method (baseline_ewma) uses a single-exponential-smoothing slope to extrapolate one frame. Neither forecasts the horizon this paper uses, so a third control is added that does: linear extrapolation, H=300 keeps the entire pipeline of this method—sliding-window smoothing, four-state classification, the slope identification factor, the FSM and the report gate—and replaces only the AR(2) recursion of Equation (4) by $x_{n+H}=x_{n}+\left(x_{n}-x_{n-1}\right)H$ over the same horizon. It therefore isolates the contribution of the extrapolation model from that of the horizon and of every other component, which is exactly the comparison that a one-frame baseline cannot provide. The results are summarized in Table 10:

**Table 10:** Method comparison results (calibration (early) segment, report = 3, relWin = 300; not a held-out evaluation).

Band	Method	Precision	F1	OWF
C	Fixed threshold	0.0486	0.0927	0.9905
	EWMA (1-frame)	0.0507	0.0965	0.9907
	Linear extrapolation, H=300	0.2042	0.3392	0.7934
	This method, state classification removed	0.2052	0.3405	0.7933
	This method (AR(2), H=300)	0.2051	0.3403	0.7933
Q	Fixed threshold	0.0009	0.0019	0.8891
	EWMA (1-frame)	0.0014	0.0027	0.8905
	Linear extrapolation, H=300	0.2483	0.3979	0.7492
	This method, state classification removed	0.2484	0.3979	0.7492
	This method (AR(2), H=300)	0.2484	0.3979	0.7492
L	Fixed threshold	0	0	–
	EWMA (1-frame)	0	0	–
	Linear extrapolation, H=300	0	0	–
	This method, state classification removed	0	0	–
	This method (AR(2), H=300)	0	0	–

Note that the two “removed” rows are component ablations of this method, run through the identical pipeline with one component switched off; they are not additional baselines. All rows use the same report gate N=3 and the same protocol relWin = 300, minLvl = 1, and are scored against the same early-segment event set. Throughout the tables, “–” marks a quantity that is undefined rather than zero. The L band needs both remarks. It has no real anomaly event in this segment, so its true-positive count is zero for every method and precision and F1 are genuine computed zeros, not missing values—the band did produce 1678 yellow warning frames (and no orange or red), so precision is 0/1678 rather than an empty ratio. Its OWF is undefined because with no event there is no relevant zone to fall outside of and is therefore marked “–”. The precision and F1 columns of this table carry the scope stated in Section 4.1: they measure how closely the warning frames of a method agree with this method’s own event list, not an independent fault detection accuracy. In particular, a physically real alarm raised by a baseline on a degradation episode that the alarm log does not turn into an event is scored as a false alarm. This affects all five rows of each band in the same way, so the comparison among them remains like-for-like, while the absolute values are not an independent accuracy. The baseline methods have extremely low precision because they lack the warning pipeline as a whole rather than any single stage of it. The Q band fixed threshold is only 0.0009 and EWMA only 0.0014, and the massive warnings they produce almost all fall in the fault-free relevant zone (OWF as high as about 0.89). Even though EWMA introduces single-step slope extrapolation, its precision is still of the same order as the fixed threshold. This method’s precision is 1–2 orders of magnitude higher than the baseline. The C band rises from 0.0486/0.0507 to 0.2051 (about 4 times), the Q band rises from 0.0009/0.0014 to 0.2484 (about 170–280 times, nearly 2.5 orders of magnitude), and the out-of-zone warning-frame fraction is also substantially lower (C 0.7933, Q 0.7492 vs. baseline 0.89–0.99). The ablations below attribute this gain to the pipeline as a whole—in particular to the smoothed trend gate, the state-conditioned threshold and the report gate that all five “this method” rows share—and explicitly not to the length of the extrapolation horizon.

Speed, the fourth dimension of the comparison. Speed is a separate criterion from the three above, and on it the ordering is different from, and less favourable to, this method. The fixed-threshold and EWMA baselines do a constant amount of work per frame, O(1), whereas this method and the same-horizon linear control both roll a recursion forward H=300 steps and are O(H). The measured per-frame cost of this method is about 1.8 μs, i.e., about $5.6\times 10^{5}$ frames per second, which is reported in Section 4.5 together with the hardware environment, the measurement protocol and the margin of about six orders of magnitude against the sampling interval of Table 6. We did not measure the absolute per-frame cost of the baseline programs, so no corresponding figures are given in this table; the comparison here is therefore one of complexity, and the single measured figure is the one for the proposed method.

What actually drives the improvement. The same-horizon control settles a question that the one-frame baselines cannot. Replacing the AR(2) recursion by a linear extrapolation over the identical horizon changes the metrics only marginally: precision 0.2051 to 0.2042 on C and 0.2484 to 0.2483 on Q, F1 0.3403 to 0.3392 and 0.3979 to 0.3979, OWF 0.7933 to 0.7934 and 0.7492 to 0.7492. The pre-warning window recall is 0.7867 on C for both and 0.9947 against 0.9933 on Q. The gain of this method over the fixed-threshold and EWMA baselines is therefore not attributable to the choice of extrapolation model. Nor is it attributable to the length of the forecast horizon, and the horizon ablation of Table 11 was run to settle that second question directly. The full pipeline was re-run with only the extrapolation horizon changed, H=1,10,50,100,300, and nothing else—same smoothing window, same online four-state classification, same trend gate, same report gate, same early segment and same labels. Event-level precision and F1 are invariant to *H* to within $3\times 10^{-4}$ on C (0.2052 at H=1 against 0.2051 at H=300) and $2\times 10^{-4}$ on Q (0.2484 at both ends), the OWF does not move beyond $3\times 10^{-4}$, and the orange, red and hard fault recalls are identical across all five columns. The only quantity that changes monotonically is the pre-warning window recall on Q, from 0.9893 at H=1 to 0.9947 at H=300, i.e., a longer horizon buys a slightly earlier first warning rather than a more precise one. The frame-level decisions do differ—0.161% of frames on C, 6.02% on Q and 0.205% on L change level between H=1 and H=300—so the horizon is not a no-op, but the changes are near-symmetric, the resulting alarm clusters are essentially the same and so are the event-level metrics. A one-step forecast thus already reproduces almost the whole performance of the H=300 configuration.

Since neither the extrapolation model nor the horizon accounts for the gap, and since the state classification moves event-level precision by about $10^{-3}$ as well, the improvement is not attributable to any one of the parts that can be switched off in isolation. We therefore state it, as the referee suggests, as a property of the pipeline as a whole. The gap comes from the combination of the smoothed trend gate that requires a rising rather than merely high $T_{2}$, the threshold placed at $T_{2}^{\mathrm{NORM}}+k\sigma$ rather than at the nominal band bound used by the fixed-threshold baseline, the consecutive-frame report gate, and the four-state context that supplies the red level. We no longer claim that forecasting 300 frames instead of one is the source of the precision gain; the horizon ablation rules that out. We also record as a limitation that the trend gate and the threshold level are not switched off individually in any of our controls, so their separate contributions remain unquantified. What the controls do establish is that the three parts that can be isolated—extrapolation model, horizon and state classification—each contribute at most a few $10^{-3}$ at event level.

This is consistent with Table 2, where the two rules differ by a factor of 4–6 in h=300 forecast error yet produce almost the same warning decisions. This is because the orange decision compares the forecast against a threshold and is dominated by whether the trajectory crosses it rather than by how accurately its endpoint is predicted. The contribution of AR(2) should therefore be stated as a robustness property—a bounded, damped forecast whose error saturates instead of growing with *h* inside the fitted regime, though not outside it (Table 3)—rather than as the source of the precision gain, and we do not claim otherwise.

Note that *H* is the number of frames over which the AR(2) recursion of Equation (4) is rolled forward; the orange condition compares the resulting ${\hat{x}}_{n+H}$ with $T_{2}^{\mathrm{NORM}}+k\sigma$. Orange recall, red recall and hard fault recall are 1.0000 on C and Q and identical in all five columns. The L band has no anomaly event in this segment; its 1678 yellow warning frames are identical in all five columns, and its precision is therefore 0/1678 throughout.

The second ablation, in which the four-state classification and the FSM legality check are switched off while everything else is kept, is equally unhelpful for the precision figures—0.2052 against 0.2051 on C and 0.2484 against 0.2484 on Q, with the OWF unchanged—but it is decisive for the hard fault. The single RED event of the C band, which the full method recalls, is missed entirely (hard fault recall 1.0000 to 0.0000). Red is produced by the FSM catching an illegal NORMAL–AMBIENT jump, and that mechanism exists only because a state sequence exists; removing the classification removes the only route by which a red warning can be raised at all. The two ablations, together with the horizon scan of Table 11, therefore attribute the improvement as follows. The parts that can be switched off in isolation each move event-level precision by at most a few $10^{-3}$: the choice of extrapolation model by about $10^{-3}$, the forecast horizon by at most $3\times 10^{-4}$, and the state classification by about $10^{-3}$. The state classification is nevertheless not optional because it is the sole source of the red level. The one-to-two-order gain over the fixed-threshold and EWMA controls is therefore a property of the pipeline as a whole rather than of any single component, and in particular not of the longer forecast. This is the honest reading of Table 10 and Table 11. The three methods’ precision values for the L band are all 0 because the L band alone has no real anomaly event in the early segment. That segment contains real anomaly events—75 on the C band and 748 on the Q band—and it is only the L band for which the count is zero so that its relevant zone is empty (see the header of Section 4.2). This is a property of the L-band record rather than a failure of the method, and the L band is therefore not included in the horizontal comparison. Note also that the early-segment and held-out-segment event sets are disjoint by construction, being a partition of the full-record events at the dividing date (C: 75+267=342; Q: 748+616=1364). The early segment is not fault-free, it simply does not contain the faults of the later segment. Note that the baseline threshold is not differentiated by band (fixed absolute temperature boundary), which is its inherent setting, and this also provides indirect evidence that this paper’s practice of calibrating four-state centers and AR(2) coefficients by band is indispensable for reducing false alarms.

Figure 4 restates Table 10 in logarithmic scale. The two left columns (C and Q) show that both the fixed threshold and EWMA baselines are pressed against the bottom for precision and F1, while this method stands out by 1–2 orders of magnitude. The OWF panel (right column) shows the opposite pattern—the baselines sit near 0.9 while this method drops to about 0.75–0.79. The visual gap mirrors Table 10 and makes the core message of this section immediate: trend extrapolation plus four-state context, not absolute-threshold crossing, is what suppresses false alarms on year-round sparse-fault monitoring.

### 4.5. Operational Performance

The test environment is a single machine with a 24-core Intel i9-13900K and 128 GB DDR5 memory. A single process serially processes the full three-band data: the average per-frame processing is about 1.8 μs, with a throughput of about $5.6\times 10^{5}$ frames/s. The full L/C/Q elapsed times are 3.4 s, 25.5 s, and 7.6 s, respectively (the above are single measurement results, affected by CPU load, cache state, and disk I/O, with about ±7% fluctuation in repeated runs, stable in order of magnitude). The algorithm is mainly based on a sliding window and incremental updates. Its per-frame complexity is O(H)—constant with respect to the length of the history but not with respect to the forecast horizon (Section 2.6)—and with H=300 fixed it is a constant in deployment, easily parallelized and extensible to multi-channel, multi-device real-time condition-monitoring deployment.

Against what reference the figures should be judged. No certified real-time standard applies to this deployment, and we do not claim compliance with one. The target is a monitoring front end that raises graded warnings for operators, not a safety-instrumented control loop that must act within a bounded response time. The requirement that does apply is the timing budget set by the application itself—a monitoring method is fast enough if it consumes a frame well within the interval at which the next frame arrives. Table 6 gives that interval, 62 s on L, 10 s on C and 34 s on Q, so even on the fastest band the per-frame budget is of the order of $10^{7}$ μs, against a measured cost of 1.8 μs. The margin is therefore about $5.6\times 10^{6}$, i.e., six to seven orders of magnitude. It is equally wide in aggregate. The three bands together generate only about 1/62+1/10+1/34≈0.15 frames per second, against a measured single-process throughput of $5.6\times 10^{5}$ frames per second, again a margin of about six orders of magnitude.

Comparison with the baselines. Compared to the baseline methods, the proposed method is more expensive. The fixed-threshold and EWMA baselines do a constant amount of work per frame, O(1), whereas the proposed method rolls the AR(2) recursion forward H=300 steps and is O(H). The claim of real-time capability therefore does not rest on being faster than the baselines (it is not) but on the absolute cost staying far below the sampling interval even after the rollout. Two caveats qualify this conclusion. First, the measurement was taken on a desktop-class processor rather than on the embedded front end that is the eventual deployment target, so the absolute microsecond figure will be worse there. The conclusion is nevertheless insensitive to the platform because a margin of six orders of magnitude would absorb an embedded front end three orders of magnitude slower and still leave the per-frame cost about three orders of magnitude below the sampling interval. Second, the ±7% run-to-run fluctuation noted above matters only for the exact figure, not for a margin of this size. We therefore state that the method meets the real-time requirement of this application with a very large margin, without claiming a certified real-time guarantee.

### 4.6. Temperature Trajectory and Three-Level Warning Visualization

**Figure 5:**
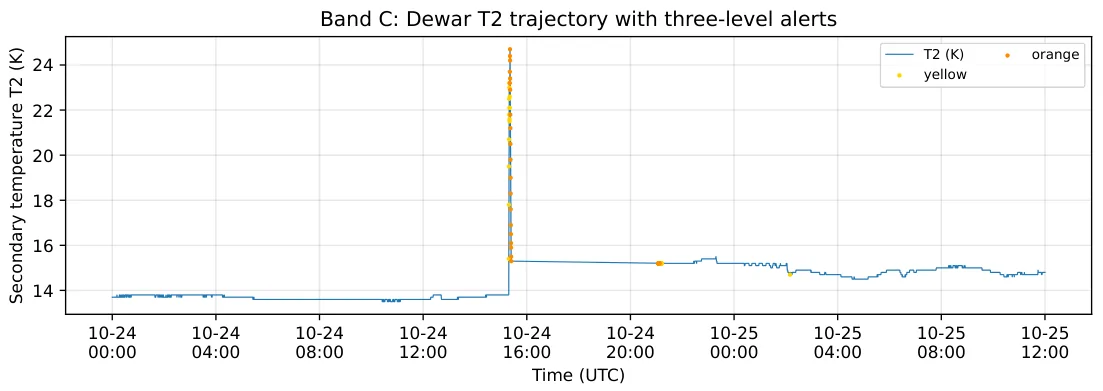
C-band fault window: secondary temperature trajectory (blue line) and yellow/orange/red three-level warning annotations (points). Warnings appear before the temperature clearly exceeds the limit, reflecting precursor capture.

**Figure 6:**
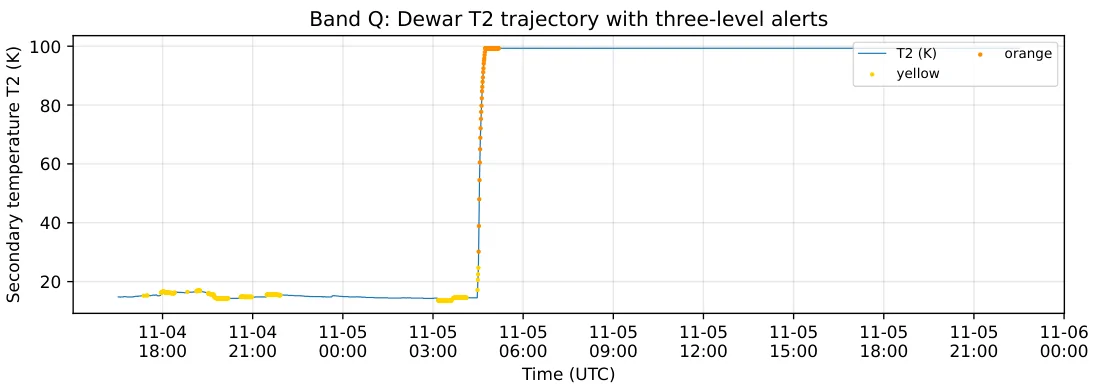
Q-band fault window: secondary temperature trajectory and three-level warning annotations. The Q band has a high bad-point rate, but the trend can still be stably perceived after smoothing and filtering.

Figure 5 and Figure 6 give the real temperature trajectories and three-level warning annotations of typical fault windows for the C/Q bands. The unified reading method is as follows. The horizontal axis is the frame index (time), the vertical axis is the secondary temperature $T_{2}$ (in K), the blue curve is the smoothed temperature trajectory, and the yellow/orange/red scattered points correspond to the yellow (early trend), orange (precursor), and red (hard fault) three-level warnings defined in Section 2.5. The two figures jointly confirm: (1) the temperature slow-rise segment is gradually captured by yellow and orange warnings, and the warnings all appear before the temperature clearly exceeds the limit, intuitively reflecting the method’s trend perception and precursor capability, consistent with the pre-warning window recall in Section 4.1 (C 0.9663 at N=1, Q 0.9968 at N=1); (2) the red level (if present in the figure) corresponds to the abrupt temperature change or the ambient jump after loss of vacuum, consistent with the FSM direct-jump judgment in Section 2.5. The difference between the two figures lies in data quality. The C band curve is smooth with clear warning boundaries. The Q band has a high original bad-point rate, but after filtering and smoothing in Section 3.3, the trend and three-level warning can still be stably perceived, indicating the method’s robustness to noisy data. Note that the windows in the figures are enlarged typical fault fragments with limited horizontal span, intended to show the correspondence between warnings and temperature evolution, rather than full-time statistical results.

### 4.7. Vacuum Auxiliary Dimension (L Only)

The L-band raw data contain a vacuum column vac (the C/Q bands do not provide this column due to sensor configuration differences, so this section only takes the L band as the object). The loss-of-vacuum frames (vac>1.0) total 13,631 frames, converted by the L band’s about 62 s sampling interval to about 234 h, accounting for 0.718% of the total monitoring duration, which is a typical long-tail sparse anomaly. After interval merging (adjacent loss-of-vacuum frames with interval less than the merge threshold are merged into segments), 9 continuous segments are obtained, with an average of about 1514 frames (about 26 h) per segment, and the longest segment corresponds to a continuous heat-leak process. Among them, 100% of the loss-of-vacuum segments are simultaneously accompanied by a secondary temperature $T_{2}>20$ K warming. Co-occurrence this strong establishes that the two channels are linked, but it does not by itself establish which moves first, so we measured the lag rather than assuming it. For each of the nine segments we located the last upward crossing of $T_{2}$ above 20 K at or before the frame at which vac first exceeds 1.0 and took the difference. The measured ordering is as follows: In 8 of the 9 segments, the temperature crossing precedes the vacuum crossing by 4 to 1158 frames (median 7 frames, about 7 min at the 62 s sampling interval). In the ninth, the secondary temperature was already at 156.0 K when vac crossed 1.0, having remained above 20 K throughout the preceding interval. Stated differently, at the instant vac crosses 1.0, the secondary temperature is already between 36.3 K and 156.0 K in all 9 segments—far above both the 20 K criterion and the ≈13 K steady-state value. At the moment $T_{2}$ crosses 20 K, the vacuum reading is still 0.005 in 6 of the 8 measurable segments (0.153 and 0.172 in the remaining two), i.e., still well below the loss-of-vacuum threshold. We therefore do not claim that the drop in vacuum precedes the temperature rise. At the thresholds and the 62 s resolution available here, the temperature channel is observed to respond no later than the vacuum channel, and the observed ordering further depends on where the two thresholds are placed, so no causal direction is asserted. What the 100% co-occurrence does support is a weaker but still useful statement: a vac anomaly reliably indicates that the warming in progress is a loss-of-vacuum event rather than, for instance, a refrigeration stoppage. The vacuum dimension is accordingly used to corroborate and disambiguate, not to gain lead time: when the temperature warning and the vacuum anomaly co-occur, the confidence of the warning can be raised and isolated noise false alarms suppressed. The suggestion that the vacuum channel could issue an alarm in advance of the temperature channel and thereby shorten the pre-warning window is not supported by these measurements, and no such claim is made here. It should be pointed out that in the real-time three-band case of Section 4.8, only the L band has a vacuum sensor and maintains a steady-state 0.005 Pa throughout, showing no sub-threshold deterioration. The C/Q bands have no vacuum sensor (reading −1), and this dimension cannot be implemented. This shows that the sensitivity conclusion of the vacuum dimension mainly relies on the offline loss-of-vacuum statistics in Section 4.7 and needs to be interpreted prudently in the real-time monitoring segment in combination with the band sensor configuration.

### 4.8. Three-Band Real-Time Monitoring Case Study

To verify the end-to-end usability of the method in a real operating environment, this paper simultaneously conducted continuous real-time monitoring on the L, C, Q band dewar monitoring data. The real-time monitoring program takes the rolling updated monitoring data file as an input and outputs to each band’s alarm log in a frame-by-frame manner. At the same time, an independent sliding-window pattern recognition analysis program (a different algorithm) performs cross-interpretation of the same log, forming a reviewable two-way verification. The three-band measurements are all in their respective steady-state or mild-trend segments, with no red/orange hard faults triggered. The real morphology and the exposed calibration problems are summarized by band below.

**Figure 7:**
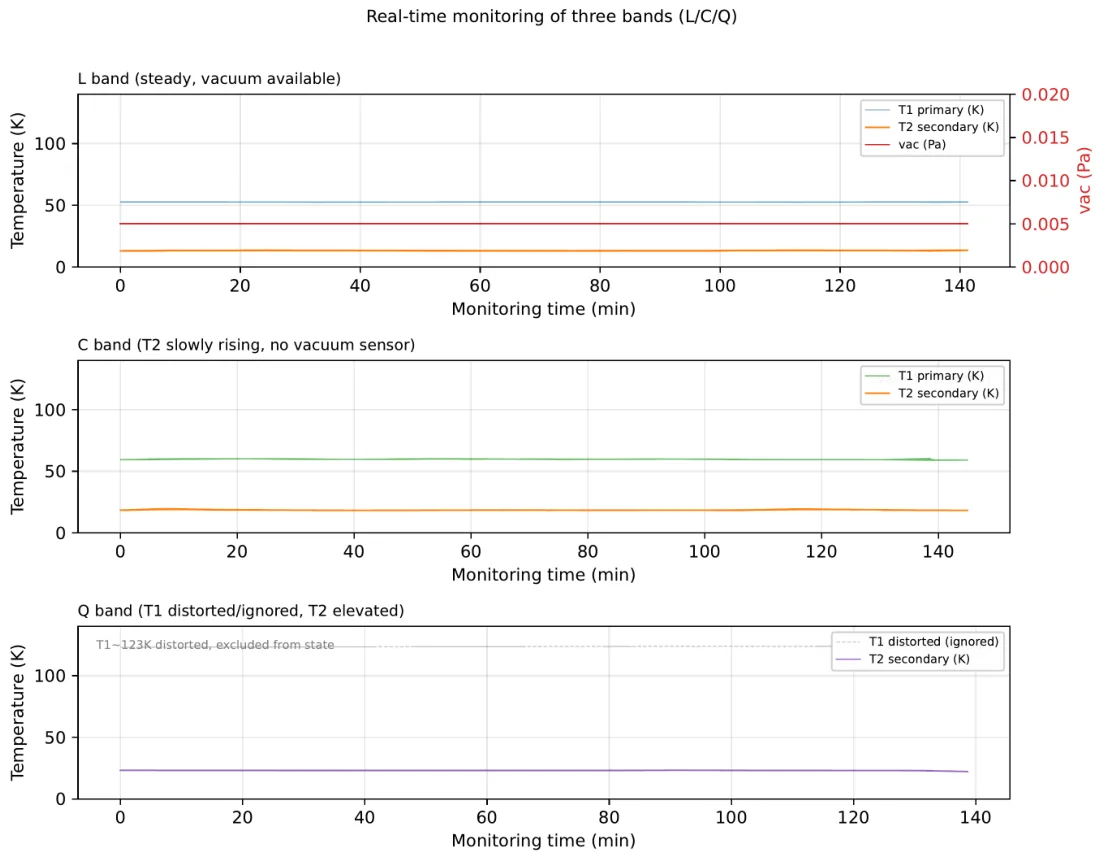
Real curves of three-band real-time monitoring (L/C/Q). Top panel: L band completely steady, $T_{2}$ about 13 K, vac maintained at 0.005 Pa; middle panel: C band $T_{2}$ slowly rises from 18.3 K to 19.6 K, state = RISE; bottom panel: Q band $T_{1}\approx 123$ K is sensor distortion (gray dashed line, state judgment ignored), $T_{2}$ about 23 K elevated. Curves drawn from the three-band real-time alarm logs, consistent with the text.

L band: completely steady. During monitoring, $T_{1}$ is stable in 52.5–52.8 K, $T_{2}$ is stable in 12.9–13.6 K, the internal temperature state is always 0 (NORMAL), and the warning level is mostly 0. Only a few frames where $T_{2}$ slightly rises to 13.5 K trigger yellow (level = 1), which is a typical steady-state condition (see the top panel of Figure 7). The vacuum vac remains constant at 0.005 Pa throughout, showing no sub-threshold deterioration, contrasting with the “long-tail sparse anomaly” in the offline loss-of-vacuum statistics of Section 4.7—this real-time segment happens to fall within the healthy main distribution.

C band: secondary temperature slow rise. $T_{1}$ is stable in 58.9–60.4 K, $T_{2}$ slowly climbs from 18.3 K to 19.6 K, the internal temperature state is always 1 (RISE), but the warning level is mostly 0, with only individual frames being yellow (see the middle panel of Figure 7). This indicates that the secondary temperature has shown a clear warming trend (reflected by state), but the instantaneous slope and extrapolation criterion did not upgrade the warning because the slope did not continuously cross the threshold, which is consistent with the interpretation intent of “early trend needs higher attention”. This band has no vacuum sensor (vac=−1), and the third dimension is invisible.

Q band: primary temperature distortion, secondary temperature elevation. The $T_{1}$ reading is constantly 123.0–123.9 K, confirmed by calibration to be a sensor distortion value (not a real physical temperature), so it is ignored when judging the state, and only $T_{2}$ is used as the interpretation basis. $T_{2}$ is stable in 22.2–23.3 K, in an elevated steady-state band. The independent analysis program judges it as “secondary temperature elevated (attention)” accordingly (see the bottom panel of Figure 7, the gray dashed line is the ignored distorted $T_{1}$). This band also has no vacuum sensor (vac=−1).

The measured exposure of state/level calibration inconsistency. The most noteworthy finding in real-time monitoring is the inconsistency between the internal temperature state (state) and the warning level (level) scales. According to the definition in Section 2.5, state is determined by the online k-means nearest-neighbor classification, indicating the temperature zone that the current temperature is in 0 = steady-state(NORMAL), 1 = rising(RISE), 2 = dropping(DROP), 3 = ambient(AMBIENT). The level is determined by instantaneous slope + AR(2) extrapolation + FSM, indicating the triggered warning level: 0 = none, 1 = yellow(early trend), 2 = orange(precursor), 3 = red(hard fault). The two are calculated independently. This inconsistency is fully reproduced in the Q-band measurement. Here the distorted $T_{1}$ value is classified into the high-level DROP/AMBIENT zone (state ≥ 2), while the $T_{2}$ slope is stationary, so the extrapolation + FSM did not upgrade the warning. The independent analysis program therefore found statistically that 30/30 frames in the window all exhibit the situation of “state already classified as dropping/ambient (state ≥ 2) but level still shows no warning (level = 0)”. The C band with state = RISE (state = 1) and level = 0 belongs to the same type of light inconsistency.

The mechanism is as follows: The nearest-neighbor classification of state only looks at which current temperature zone it falls into, while level depends on whether the instantaneous slope continuously crosses the threshold. When the temperature is in a high-level platform or distorted high level, the per-frame instantaneous slope alternates between the rising edge and the falling edge (or distorted constant value). The falling-edge/constant segment slope is classified into the DROP zone or maintains a low slope, and the extrapolation + FSM did not upgrade the warning level because the slope did not continuously cross the threshold, so level remains 0. This shows that the temperature state has reached a high level (reflected by state), but the warning level missed the report because it depends on the instantaneous slope. This phenomenon is visible in all three L/C/Q bands (L is mostly state = 0 so it is not obvious, while C/Q clearly expose it), proving that this is a design defect rather than an occasional occurrence.

Improvement directions. (1) In addition to referring to the instantaneous slope, level upgrade should also incorporate the absolute temperature-zone threshold—when state has fallen into high-level zones such as dropping/ambient (state ≥ 2) and the absolute temperature exceeds the limit, at least a yellow warning should be maintained, to avoid the “state exists but no level” miss; (2) the operation side should read both state and level, and cannot judge safety solely by level = 0, but should take the internal temperature state as the final basis; (3) when a band’s primary temperature reading is distorted (such as Q band’s $T_{1}\approx 123$ K, far above the physical NORMAL bound $T_{1}^{\mathrm{norm}}=54.0$ K of that channel; the training-period NORMAL center of Q-band $T_{1}$ is itself 177.29 K, also above that bound, so the channel is distorted across its whole range rather than at one reading—the value of about 18 K often quoted as the steady-state center of this channel is therefore misleading, 18 K being the $T_{2}$ center), distortion detection and elimination should be done before state judgment (this paper has adopted the temporary strategy of ignoring $T_{1}$ and using only $T_{2}$ for the Q band), suggesting that the online center needs boundary constraints outside the distribution or the introduction of absolute physical thresholds as a fallback.

Effect of the state/level inconsistency on the reported tables. The inconsistency is not confined to the real-time case study. Every warning level in Table 7, Table 8, Table 9 and Table 10 is produced by the same decision logic, so the same “state but no level” situation is present throughout the evaluation. On the C band, 2,140,334 of 7,826,414 early-segment frames (27.3%) carry state ≥1 with level =0, and 937,277 (12.0%) carry state ≥2; on the Q band the figures are 1,524,861 of 2,372,634 (64.3%) and 357,867 (15.1%); on the C held-out segment the figures are 4,026,446 of 6,345,108 (63.5%) and 857,581 (13.5%). None of these frames is counted as a warning under the present protocol, so all numbers reported in this paper are conditional on it.

Fixing it is not free, and we quantify the cost rather than assert it. Implementing improvement direction (1)—holding at least a yellow warning when state ≥2—and re-running the evaluation changes every table. On the early segment with report =3, C-band precision falls from 0.2051 to 0.0156 and F1 from 0.3403 to 0.0308, with the warning cluster count rising from 17,404 to 328,130; on Q, precision falls from 0.2484 to 0.1488 and F1 from 0.3979 to 0.2591, with clusters rising from 92,502 to 201,390. On the held-out segment the same change moves precision from 0.1146 to 0.0484 and F1 from 0.2056 to 0.0924. Recall stays at 1.0000 throughout because it is already saturated. The change is not purely detrimental: precursor-window recall rises from 0.7867 to 0.8800 on the early C segment and from 0.9513 to 0.9700 on the held-out C segment, so the floor does make the method earlier. The trade is precision against earliness, which is a genuine design choice rather than a defect that can be removed for free; we therefore report the present numbers under the deployed protocol and list the floor as an unadopted improvement rather than applying it silently.

Effect of the Q-band $T_{1}$ override. The Q band’s $T_{1}$ channel is distorted, and the deployed configuration excludes it from state classification (useT1State = 0), judging state on $T_{2}$ alone. This applies to every Q-band number in Table 7, Table 8, Table 9 and Table 10, and it deserves the same scrutiny as the inconsistency above—but the answer is different: the override does not change any reported number. Forcing $T_{1}$ back into the classification leaves the state distribution unchanged (802,569/1,194,045/373,469 frames in states 0/1/2 on the early segment, identical to the deployed configuration) and reproduces precision 0.2484, F1 0.3979 and OWF 0.7492 exactly. The reason is that the incremental center update carries the DROP center to $T_{1}=122.5$ K, close to the distorted reading of about 123 K, so the assignment is the same either way; and the level decision does not depend on state. The override is thus a nominal safeguard at the present protocol. It would become material if the state floor above were adopted, and it does not remove the need for the distortion detection of improvement direction (3).

The above real measurement case proves that this method can not only reproduce the evaluation results offline but also continuously monitor on real operating data in a three-band parallel manner. The cross-validation mechanism can independently expose algorithm design defects (state/level inconsistency, sensor distortion), providing a clear direction for subsequent iteration.

## 5. Discussion

Data-label characteristics and metric interpretation (candid note). This dataset lacks independently labeled hard fault timestamps; the real anomaly events used as the evaluation baseline are extracted from the continuous warning segments of the alarm log and therefore share the same source as the warnings. A partial cross-check of a subset of these labels against the raw sensor record is reported in Section 4.1. It confirms that the labeled events are physically real, but it does not supply the independent fault timestamps that are still missing here. Therefore, (1) recall-type metrics (including the structural upper bound of main-evaluation recall and pre-warning window recall) are high and cannot be used as strict evidence of “independent fault capture rate”; (2) the core contribution evidence of this paper should fall on precision/false alarm control and relative improvement over the baseline—under the same event definition, this method’s warning cluster precision is 1–2 orders of magnitude higher than the baseline (see the baseline comparison in Section 4.4), which is the substantive embodiment of the method’s validity. The same reservation that applies to recall applies to the precision and F1 values as well. They measure agreement with this method’s own event list and are therefore not measures of independent fault detection accuracy either. An equally real alarm that the alarm log does not contain is scored as a false alarm and the method that defines the events is favoured by construction (see the scope statement of Section 4.1). The relative ordering obtained under one and the same scoring protocol is what survives this reservation. Future work needs to introduce independent fault ground truth (such as equipment maintenance records) to strictly measure the lead time.

L band has no orange/red warning. This is not an algorithm defect but a real data characteristic. The L band AR(2) coefficients $a_{1}+a_{2}=0.8483<1$ (stationary convergence), and the extrapolated steady-state value is $c/\left(1-a_{1}-a_{2}\right)=1.8448/\left(1-0.3881-0.4602\right)\approx 12.16$ K, lower than the orange threshold $T_{2}^{\mathrm{norm}}+2\sigma =12.5+2\times 0.1193=12.739$ K (Table 4), so the extrapolated value is mathematically always below the threshold, and orange must be 0. Red is only triggered by FSM illegal jumps, and neither the front nor back segment of L has such hard jumps. This provides indirect evidence that the L band was in a good steady state during the observation period. It should be noted that the above three nearby but distinct quantities arise and should not be confused. They are the AR(2) stationary value $c/(1-a_{1}-a_{2})\approx 12.16$ K, the NORMAL cluster center $c_{0}=12.69$ K of Table 5, and the physical NORMAL upper bound $T_{2}^{\mathrm{norm}}=12.5$ K that the threshold actually uses. They differ because they are computed on different bases—a fitted stationary limit, a state-conditioned sample mean, and a fixed physical bound—and the spread among them is 0.53 K (12.16–12.69 K), about twice the margin 2σ=0.24 K, which is exactly why Table 4 states all of them explicitly. The decisive quantity is the AR(2) stationary value 12.16 K, which lies 0.58 K below the orange threshold 12.739 K, while the other two values fall in between. The conclusion that the L band cannot produce an orange warning is therefore robust to this ambiguity. Had the cluster center 12.69 K been used in place of the physical bound, the threshold would have been 12.69 + 0.239 = 12.929 K instead, and the conclusion would be unchanged.

Method advantages and limitations. The advantages include white-box interpretability, precursor capability through trend extrapolation, reduction of false alarms through state adaptation, and good real-time performance. The limitations are as follows: (1) the precision absolute value is still affected by the sparsity of real faults (warning clusters mostly fall in the fault-free relevant zone); (2) the L band produced no high-level warning due to data stationarity and needs validation on more fault samples; (3) same-source labels cause recall-type metrics to be high and need independent ground truth supplement; (4) of the parameters (W,H,k,θ,δ,N) only the report gate *N* is selected by ablation—W=50, H=300, k=2, θ=0.01 K/frame and δ=0.005 K/frame are implementation defaults and because the deployed θ>δ the dead-zone is inert at this setting. The θ–δ interaction is unexplored (raising δ above θ raises C-band precision from 0.2051 to 0.4542 but lowers recall from 1.0000 to 0.8000), so cross-device generalization needs recalibration.

Comparison perspective with existing work. Compared with SPC control charts, this paper introduces online k-means multimodal modeling and AR(2) physical extrapolation, making the warning have both “state semantics” and “trend precursor”. Compared with black-box machine learning, this paper has a transparent mechanism, requires few parameters, and is auditable, making it better suited to high-reliability scenarios.

## 6. Conclusions and Future Work

Conclusion. This paper casts the problem as sensor-based real-time condition monitoring and fault pre-warning of cryogenic dewar systems and takes the real multi-sensor monitoring data of the L, C, and Q band cryogenic dewars of a 26 m radio telescope as a representative case study. The proposed online k-means + AR(2) extrapolation + FSM three-level warning framework, on real three-band monitoring data (over 20 million frames, including a bad-point rate as high as 37.5%), produces warnings with the real threshold applied by the program running parameters. It improves precision by 1–2 orders of magnitude over the fixed-threshold/EWMA baseline, achieves a pre-warning window recall of 0.93–1.00, and has a real-time throughput of about $5.6\times 10^{5}$ frames/s. All experimental numbers are obtained by end-to-end reruns on real data, with no simulation on the evaluation side.

Future work. (1) Introduce independent fault ground truth. At present, the fault labels are extracted from the same alarm log that also generates the warnings, so the recall-type metrics carry an upward bias that only independent maintenance and shutdown records can remove. With such records, lead time can also be measured strictly; (2) extend the framework to multi-channel, multi-device distributed real-time deployment, using multi-core parallelism to further reduce end-to-end latency; (3) study automatic cross-band/cross-device parameter calibration methods; (4) fuse multiple physical quantities such as vacuum to build joint warnings, improving the detectability of rare faults (such as the L band); (5) revisit batch anomaly detectors such as the isolation forest and its minimum spanning tree variant [31,32] as candidates for offline retrospective analysis of archived records, where the complete history is available and the constraints that exclude them from the online loop no longer apply.

## Figures and Tables

**Table 1:** Main symbol definitions.

Symbol	Meaning
*W*	Sliding-window length (frames), 50
*n*	Current frame index
$x_{n}$	Raw secondary temperature of frame *n*
${\bar{T}}_{2}\left(n\right)$	Smoothed secondary temperature of frame *n*
${\bar{T}}_{1},{\bar{T}}_{2}$	Mean of the primary and the secondary temperature over the window
$c_{s}$	Online k-means cluster center $\left(T_{1},T_{2}\right)$ of state *s*, physically initialized
ρ	Online incremental-update learning rate, 0.01
$a_{1},a_{2},c$	AR(2) coefficients
σ	Residual standard deviation of the AR(2) model, 0.0715 K on C
*H*	AR(2) extrapolation horizon (frames), 300 (5.17 h on L, 50 min on C, 2.83 h on Q)
*k*	Orange threshold multiplier, 2
θ	Minimum trend-slope threshold, 0.01 K/frame
δ	Slope dead-zone, 0.005 K/frame (inert at this setting, see Section 2.5)
*S*	State-stability frames required by the red decision, 5
*N*	Consecutive-frame gate (report gate), main value 3
relWin	Relevant-zone forward frames (pre-warning window), main value 300
postWin	Relevant-zone backward frames (post-event window), main value 50

**Table 2:** Multi-step extrapolation RMSE (K) on the held-out segment, rolling-origin evaluation; AR(2) coefficients fitted on the pre-26 June 2024 segment.

Band	Method	h=1	h=10	h=50	h=100	h=300
L	AR(2)	0.0254	0.1118	0.1675	0.1701	0.1670
	Linear	0.0031	0.0232	0.1181	0.2355	0.6929
C	AR(2)	0.0248	0.1540	0.5380	0.7516	0.8596
	Linear	0.0129	0.1051	0.7485	1.6141	4.9919
Q	AR(2)	0.0076	0.0774	0.2968	0.4168	0.6321
	Linear	0.0012	0.0321	0.3535	0.7974	2.4204

**Table 3:** The same rolling-origin extrapolation error as in Table 2, but with the origins restricted to the RISE (warming) state, the regime in which precursor warnings are raised.

Band	Method	h=1	h=50	h=300
L	AR(2)	1.4230	9.3044	8.9631
	Linear	0.0053	1.3352	11.5630
C	AR(2)	0.0531	1.2420	2.5636
	Linear	0.0148	1.8409	11.4422
Q	AR(2)	0.0550	2.8464	14.8135
	Linear	0.0009	1.0098	6.3575

**Table 6:** Frame-to-time conversion and per-band lead time for a 300-frame window.

Band	Median Interval		300 Frames	
	Early Segment	Later Segment	Early Segment	Later Segment
L	62 s (bimodal 32 s/62 s)	62 s	2.67–5.17 h	5.17 h
C	10 s	10 s	0.83 h (50 min)	0.83 h (50 min)
Q	34 s	34 s	2.83 h	2.83 h

**Table 7:** Main evaluation results on the held-out segment (frames strictly after 26 June 2024; relWin = 300, postWin = 50, minLvl = 1).

Band	*N*	Real Anomaly	Recall	Hard Fault	Pre-Window	Precision	F1	OWF
		Events		Recall	Recall			
L	1	0	–	–	–	–	–	–
	3	0	–	–	–	–	–	–
C	1	267	1.0000	1.0000 (1/1)	0.9663	0.1373	0.2415	0.8828
	3	267	1.0000	1.0000 (1/1)	0.9513	0.1146	0.2056	0.8829
Q	1	616	1.0000	–	0.9968	0.2391	0.3860	0.7067
	3	616	1.0000	–	0.9935	0.2906	0.4504	0.7069

**Table 11:** Forecast-horizon ablation: the complete pipeline with only the extrapolation horizon *H* changed. Early (calibration) segment, report = 3, relWin =300, minLvl =1, identical labels; the H=300 column reproduces the “this method” row of Table 10.

Band	Quantity	H=1	H=10	H=50	H=100	H=300
C	Precision	0.2052	0.2056	0.2056	0.2055	0.2051
	F1	0.3405	0.3411	0.3411	0.3410	0.3403
	OWF	0.7933	0.7932	0.7930	0.7933	0.7933
	Pre-warning recall	0.7867	0.7867	0.7867	0.7867	0.7867
Q	Precision	0.2484	0.2483	0.2485	0.2482	0.2484
	F1	0.3979	0.3978	0.3981	0.3977	0.3979
	OWF	0.7492	0.7492	0.7492	0.7493	0.7492
	Pre-warning recall	0.9893	0.9920	0.9920	0.9947	0.9947

## Data Availability

The dewar monitoring data used in this study are operational data of the telescope and are available from the corresponding author on reasonable request.

## Abbreviations

The following abbreviations are used in this manuscript:

AR(2)	Second-order autoregressive model
EWMA	Exponentially weighted moving average
FSM	Finite state machine
LNA	Low-noise amplifier
SPC	Statistical process control
